# Endocytic Sorting and Recycling Require Membrane Phosphatidylserine Asymmetry Maintained by TAT-1/CHAT-1

**DOI:** 10.1371/journal.pgen.1001235

**Published:** 2010-12-09

**Authors:** Baohui Chen, Yue Jiang, Sheng Zeng, Jiacong Yan, Xin Li, Yan Zhang, Wei Zou, Xiaochen Wang

**Affiliations:** 1 Graduate Program in Chinese Academy of Medical Sciences and Peking Union Medical College, Beijing, China; 2 National Institute of Biological Sciences, Beijing, China; University of California San Diego, United States of America

## Abstract

Endocytic sorting is achieved through the formation of morphologically and functionally distinct sub-domains within early endosomes. Cargoes destined for recycling are sorted to and transported through newly-formed tubular membranes, but the processes that regulate membrane tubulation are poorly understood. Here, we identified a novel *Caenorhabditis elegans* Cdc50 family protein, CHAT-1, which acts as the chaperone of the TAT-1 P4-ATPase to regulate membrane phosphatidylserine (PS) asymmetry and endocytic transport. In *chat-1* and *tat-1* mutants, the endocytic sorting process is disrupted, leading to defects in both cargo recycling and degradation. TAT-1 and CHAT-1 colocalize to the tubular domain of the early endosome, the tubular endocytic recycling compartment (ERC), and the recycling endosome where PS is enriched on the cytosolic surface. Loss of *tat-1* and *chat-1* function disrupts membrane PS asymmetry and abrogates the tubular membrane structure. Our data suggest that CHAT-1 and TAT-1 maintain membrane phosphatidylserine asymmetry, thus promoting membrane tubulation and regulating endocytic sorting and recycling.

## Author Summary

The process by which cells take up nutrients and other large molecules from the extracellular environment is known as endocytosis. At the cell surface, external molecules become enclosed in membrane spheres called endosomes. Early endosomes serve as a sorting station, directing the contents (cargo molecules) to the correct compartment within the cell. This is thought to be achieved by the formation of membrane structures with distinct shape and function. For example, cargoes destined for recycling and degradation are processed through tubular membrane structures and big vesicular compartments, respectively. However, it is poorly understood how early endosome membranes are shaped into different structures. Here we show that two proteins, CHAT-1 and TAT-1, regulate membrane structure and are important for normal endocytic transport in the nematode worm *C. elegans*. TAT-1 and CHAT-1 are found in tubular membrane structures along the sorting and recycling pathway, where they enrich the outer membrane layer with a lipid called phosphatidylserine (PS) and probably change the membrane curvature. Loss of *tat-1* and *chat-1* function disrupts the asymmetric distribution of PS, abolishes tubular membrane structures, and abrogates endocytic sorting/recycling. Our data support a role of TAT-1/CHAT-1–regulated membrane PS asymmetry in promoting membrane tubulation for endocytic cargo sorting and recycling.

## Introduction

In eukaryotic cells, internalized cargoes are transported to early endosomes where they are sorted to be recycled back to the plasma membrane, degraded in lysosomes or delivered to the trans-Golgi network. Early endosomes display a complex and pleiomorphic organization with many tubular processes emanating from central vesicular elements as revealed by three-dimensional reconstruction [1]–[4]. Internalized receptors dissociate from their ligands in early endosomes which have a slightly acidic internal pH; subsequent segregation of the receptor and ligand is thought to be achieved by a geometry-based mechanism [5]. Receptors and other membrane proteins concentrate in the tubular extensions which contain most of the endosomal membrane, whereas soluble contents are enriched in the vesicular components which account for the bulk of the endosomal volume [1], [6], [7]. The recycling vesicles which arise from the tubular extensions may undergo fast recycling, by fusing directly with plasma membranes, or slow recycling, by transporting cargoes through the endocytic recycling compartment (ERC), a collection of tubular membrane structures arranged around the microtubule-organizing center [7], [8]. Both cargo sorting and subsequent recycling require extensive membrane remodeling to form tubular extensions, which have a high ratio of surface area to luminal volume, thereby effectively concentrating cargoes on recycling membranes. However, it is not clear at present how these tubular processes are formed and maintained.

Both proteins and lipids are required for shaping membranes into various structures including tubular extensions. For example, BAR (Bin/amphiphysin/Rvs) domain proteins, which are central regulators of membrane remodeling, are capable of inducing membrane tubulation [9]. Members of the EHDs/RME-1 family of ATPases, which are important regulators of endocytic recycling in mammals (EHD1-4) and *C. elegans* (RME-1), associate with vesicular and tubular membranes in vivo and tubulate liposomes in vitro [10]–[14]. On the other hand, phospholipids regulate membrane shaping by either recruiting and activating effector proteins on target membranes or directly affecting membrane curvature. For instance, membrane-shaping proteins like BAR proteins and dynamin are targeted to specific membrane compartments by binding to different phosphoinositides through either a lipid-binding domain (PH or PX) or by electrostatic interaction or both, while phospholipid-binding and membrane deformation by EHDs/RME-1 family proteins appear to be mediated through their helical domains [15], [16]. In addition to acting through a protein-recruiting mechanism, phospholipids can directly affect membrane curvature. It has been observed that addition of phosphatidylserine (PS) to ATP-containing erythrocyte ghosts stimulates the formation of endocytic vesicles [17]. Notably, phosphatidylserine is asymmetrically arranged between the two membrane leaflets, being enriched in the inner leaflet of cell membranes [18]. As the most abundant anionic phospholipid of cell membranes, PS regulates surface charge and protein targeting in cultured cells, where it is also observed on the cytosolic surface of endosomes and lysosomes [19]. However, it remains to be determined whether PS, or PS asymmetry, is involved in shaping membranes into tubular processes during sorting and recycling.

Previous studies suggest that establishment and maintenance of PS asymmetry require the activity of type IV P-type ATPase family proteins (P4-ATPases), which selectively sequester PS and phosphatidylethanolamine in the cytosolic leaflet of the membrane [17]. The P4-ATPases are a large family of putative aminophospolipid translocases with 14 members in human, 5 in yeast and 6 in *C. elegans* [20], [21]. The two founding members, mammalian ATP8A1 (ATPase II) and yeast Drs2p, were found to transfer spin- or fluorescent-labeled PS analogues from the inner to the outer membrane leaflet when purified and reconstituted into proteoliposomes, indicating that they have intrinsic flippase activity [22], [23]. Deletion of *DRS2* causes defects in phospholipid translocation and protein transport from the TGN to endosomes and vacuoles in yeast [24]–[27]. Interestingly, loss of function of yeast Cdc50p, a transmembrane protein that does not contain sequence features indicative of a direct involvement in phospholipid translocation, results in similar defects in lipid asymmetry and vesicular transport to those caused by *DRS2* deletion [28], [29]. Cdc50p and the related protein Lem3p were later found to form complexes with P4-ATPases Drs2p and Dnf1p, respectively, and were shown to facilitate transport of the complexes out of the ER [28], [29]. Moreover, a recent study suggested that Cdc50 proteins are integral components of P4-ATPases and directly participate in the ATPase reaction cycle [30]. Nevertheless, the cellular function of Cdc50 family proteins in multicellular organisms remains unknown.

In *C. elegans*, the P4-ATPase TAT-1 which is most closely related to yeast Drs2p and mammalian ATP8A1, maintains cell surface PS asymmetry, thus preventing appearance of PS in the outer leaflet of the plasma membrane [31]. Moreover, an early step of endocytosis and trafficking in the lysosome biogenesis pathway was found to be defective in *tat-1(lf)* mutants which accumulate large intestinal vacuoles with characteristics of late endosomes or lysosomes [32]. However, it is not known how these different endocytic trafficking processes are affected in *tat-1(lf)* animals, or whether the defects are caused by disruption of PS asymmetry.

In the present study, we identified a novel *C. elegans* Cdc50 family protein, CHAT-1, which acts as the chaperone of the TAT-1 P4-ATPase. Endocytic sorting is severely affected in *chat-1* and *tat-1* mutants, causing abnormal cargo recycling and degradation. CHAT-1 and TAT-1 associate with PS-coated tubular membranes of early endosomes, endocytic recycling compartments (ERCs) and recycling endosomes. Loss of *tat-1* and *chat-1* function disrupts membrane PS asymmetry and abrogates the tubular membrane structure of sorting and recycling compartments. Our data suggest that TAT-1 and CHAT-1 maintain membrane PS asymmetry to regulate membrane tubulation for cargo sorting and recycling.

## Results

### *chat-1* and *tat-1* mutants disrupt plasma membrane PS asymmetry

**Figure 1:**
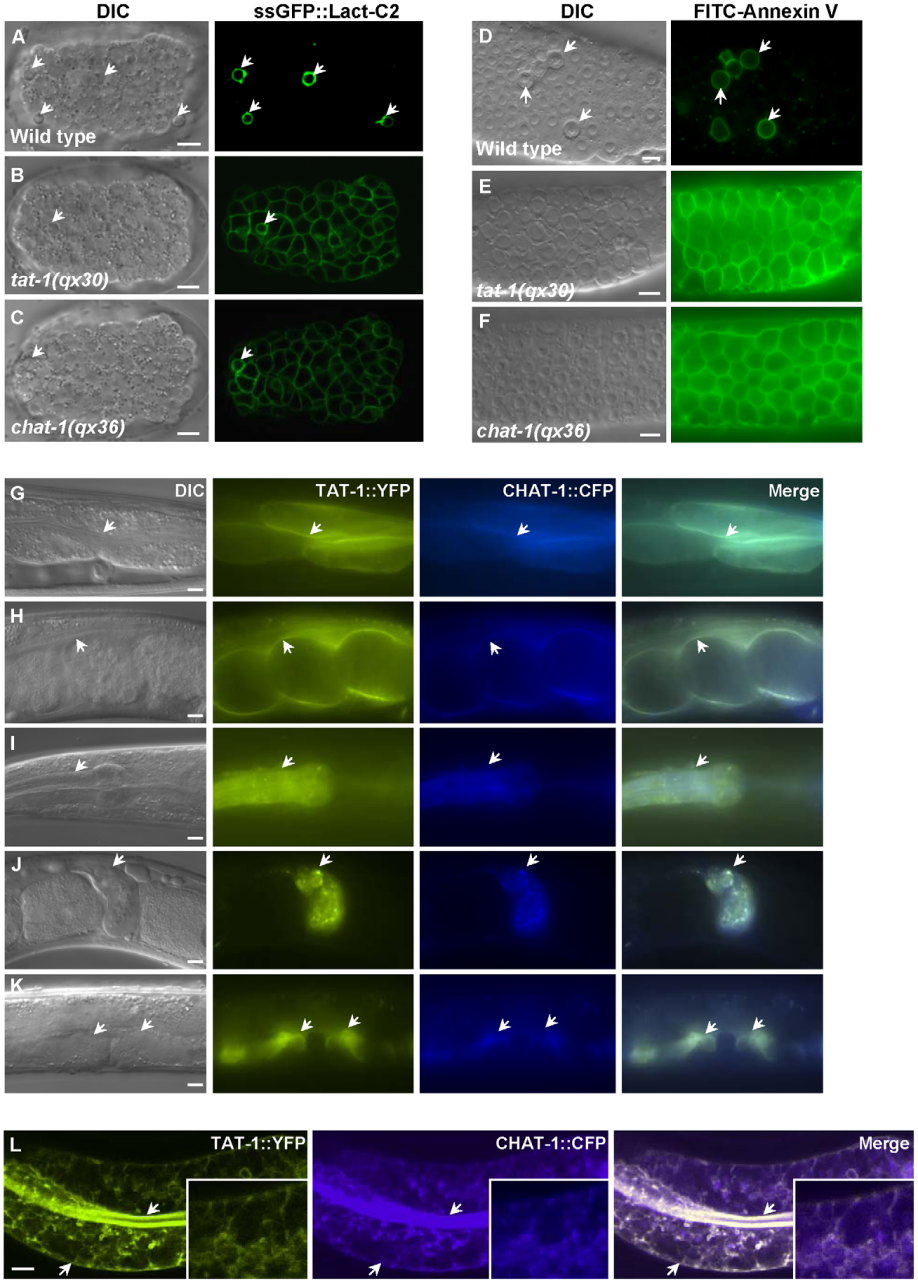
CHAT-1 and TAT-1 regulate plasma membrane PS asymmetry and express in the same tissues. (A–C) DIC and confocal fluorescent images of wild-type (A), *tat-1(qx30)* (B) and *chat-1(qx36)* (C) embryos expressing ssGFP::Lact-C2 driven by heat-shock promoters. (D–F) DIC and fluorescent images of dissected gonads stained with FITC-Annexin V from wild type (D), *tat-1(qx30)* (E) and *chat-1(qx36)* (F). Arrows in (A–D) indicate apoptotic cells labeled by ssGFP::Lact-C2 or FITC-Annexin V. (G–K) DIC and fluorescent images of wild-type animals expressing both TAT-1::YFP and CHAT-1::CFP driven by endogenous promoters. TAT-1 and CHAT-1 expression can be observed in the same cell types (arrows). (L) Confocal fluorescent images of wild-type animals expressing TAT-1::YFP and CHAT-1::CFP driven by the intestine-specific promoter *vha-6*. TAT-1 and CHAT-1 colocalize to plasma membranes (arrows) and various intracellular compartments in the intestine. Insets show an amplified view with a magnification of *X 1.8*. Scale bars: 5 µm.

PS is usually confined to the inner leaflet of the plasma membrane and only appears on the cell surface during apoptosis [33]. From a forward genetic screen for mutants which disrupt specific labeling of apoptotic cells by a PS-binding protein, we isolated the mutant *qx36*, and several alleles of *tat-1*, which encodes a *C. elegans* P4-ATPase [34] (Materials and Methods). Since P4-ATPases are known to regulate membrane PS asymmetry [17], we investigated whether PS distribution was disrupted in our mutants. Surface-exposed PS can be detected using the secreted fluorescent biosensor GFP::Lact-C2 or Annexin V, both of which bind selectively to PS [19], [31]. In wild-type animals, surface-exposed PS was only observed around apoptotic cells (Figure 1A, 1D). In *qx36* and *tat-1* mutants, PS labeling was seen on the surface of virtually all cells, indicating that plasma membrane PS asymmetry was disrupted and PS was exposed on the surface of both living and dying cells (Figure 1B–1F; Figure S1A–S1E) [31]. In contrast, no surface labeling was observed in either wild type, *qx36* mutants or *tat-1(qx30)* mutants using a secreted biosensor GFP::Lact-C2(AAA) which does not bind PS (Figure S1H–S1J) [19].

We cloned the gene affected in *qx36* mutants and found that it encodes a *C. elegans* homolog of yeast Cdc50p (Figure S2A, S2L; Materials and Methods). Since Cdc50p is a chaperone and non-catalytic subunit of Drs2p, the P4-ATPase homologous to TAT-1 [28], [30], we named this gene *chat-1* (chaperone of *tat-1*). CHAT-1 is most similar to Cdc50p in yeast and CDC50A in human (Figure S2L). All three are predicted to be integral membrane proteins containing two transmembrane domains [35]. The *chat-1* gene in *qx36* mutants contained a C to T transition which resulted in a premature stop codon after Leu 94 (Figure S2A). Similar phenotypes were also observed in *ok1681*, a deletion mutant of *chat-1*, or with RNAi inhibition of *chat-1* activity (Figure S1F, S1G, and S1K). Although 3 different alternatively spliced *chat-1* transcripts are predicted (www.wormbase.org), only *chat-1*a overexpression rescued both the PS asymmetry and membrane trafficking defects of *qx36* mutants (Figure S2A-S2G and see below).

### CHAT-1 colocalizes with TAT-1

To examine CHAT-1 expression, we generated a CHAT-1::CFP translational fusion under control of the *chat-1* promoter (P*_chat-1_chat-1::cfp*) which fully rescued the *qx36* mutant phenotypes (Figure S2A). CHAT-1 was expressed from early embryogenesis through all larval and adult stages in various cell types where TAT-1 is also expressed (P*_tat-1_tat-1::yfp*) (Figure 1G–1K; data not shown). When specifically expressed in intestine cells, CHAT-1 and TAT-1 were found to colocalize to both apical and basolateral membranes as well as various intracellular structures (Figure 1L; Figure S3A). In *chat-1(qx36)* mutant intestine, TAT-1::GFP was lost from the plasma membrane and mainly colocalized with the ER-specific marker mCHERRY::TRAM (Figure S3B, S3C). Similarly, in *tat-1(qx30)* mutant intestine, CHAT-1::GFP was completely trapped in the ER (Figure S3D, S3E). These data are consistent with previous findings that Cdc50 family proteins and P4-ATPases are mutually required for exiting the ER and that TAT-1 and CHAT-1 are predicted to function as a complex [28], [36].

### *chat-1* and *tat-1* mutants affect endocytic trafficking through early endosomes

In *chat-1* mutants, many large vacuoles accumulated in intestinal cells from early larval stages onwards, a phenotype that was described previously in a *tat-1(lf)* allele (Figure S1K, S1L; Figure S4A-S4C) [32]. Overexpression of CHAT-1 (and TAT-1) driven by either the endogenous promoter or the intestine-specific *vha-6* promoter fully rescued the vacuolation phenotype, indicating that CHAT-1/TAT-1 act cell-autonomously to regulate membrane trafficking processes (Figure S2A, S2H–S2K; data not shown) [32], [37].

**Figure 2:**
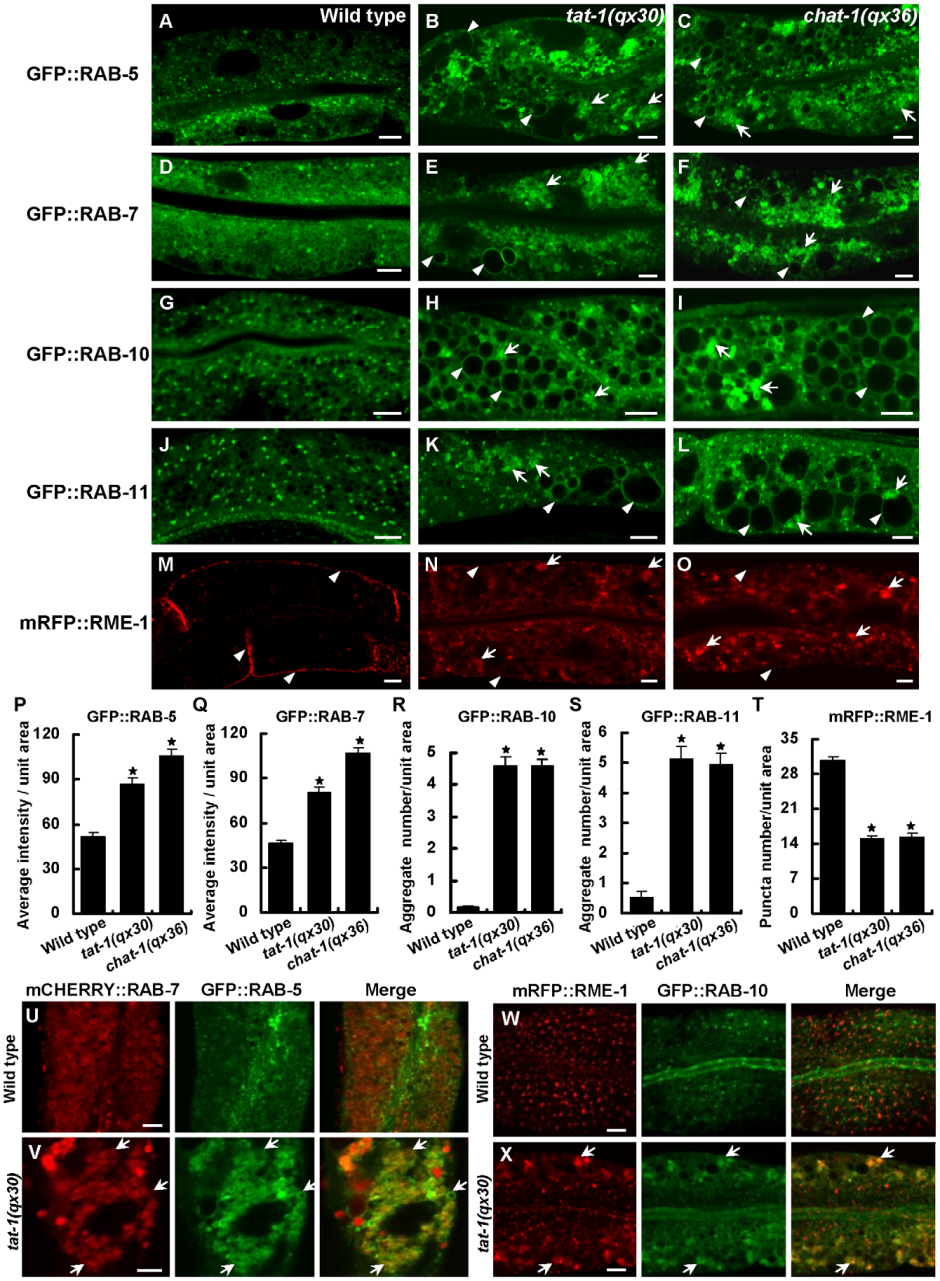
Various endocytic compartments are defective in *tat-1* and *chat-1* mutants. (A–O) Confocal fluorescent images of the intestine in wild type (A, D, G, J, M), *tat-1(qx30)* (B, E, H, K, N) and *chat-1(qx36)* (C, F, I, L, O) that express GFP::RAB-5 (A–C), GFP::RAB-7 (D–F), GFP::RAB-10 (G–I), GFP::RAB-11 (J–L) or mRFP::RME-1 (M–O). Arrowheads indicate labeling of abnormal vacuoles in mutant strains or membrane staining of mRFP::RME-1. Arrows show aggregated intracellular vesicles. (P–Q) Quantification of the average total intensity of GFP::RAB-5 (P) and GFP::RAB-7 (Q) per unit area. (R–S) Quantification of the average number of aggregated structures labeled by GFP::RAB-10 (R) and GFP::RAB-11 (S). (T) Quantification of basolateral mRFP::RME-1-positive structures in (M–O). In (P–T), data are shown as mean±SEM. *P<1.5×10^−10^. (U–X) Confocal fluorescent images of the intestine in wild type (U, W) and *tat-1(qx30)* (V, X) that coexpress mCHERRY::RAB-7 and GFP::RAB-5 (U, V) or mRFP::RME-1 and GFP::RAB-10 (W, X). Arrows indicate colocalization of RAB-5 and RAB-7 or RME-1 and RAB-10. Scale bars: 5 µm.

We next examined the distribution of various endolysosomal proteins in *chat-1* and *tat-1* mutants. GFP::RAB-5 labels early endosomes, which appeared as small punctate structures in wild-type animals but became significantly enriched and aggregated in *tat-1(qx30)* and *chat-1(qx36)* mutants (Figure 2A–2C, 2P). GFP::RAB-5 was also found on the surface of most abnormal vacuoles (Figure 2B, 2C; Figure S4D–S4F). GFP::RAB-7 labeled a small number of punctate structures and many big ring-like vesicles in wild-type intestine, which likely represent early/late endosomes and early lysosomes, respectively (Figure 2D) [38]. In *chat-1(qx36)* and *tat-1(qx30)* mutants, however, GFP::RAB-7-positive puncta significantly increased in number and formed aggregates, with a concomitant reduction of the big ring-like structures (Figure 2E, 2F, 2Q). Many abnormal vacuoles were marked by GFP::RAB-7, and some of them were positive for LMP-1::GFP, a lysosome-associated membrane protein (Figure 2E, 2F; Figure S4G-S4H, -S4R) [32]. The RAB-7 aggregation was also observed in *tat-1* and *chat-1* mutants stained with anti-RAB-7 antibodies (Figure S5A–S5C). In *tat-1(qx30)* mutants, RAB-5 and RAB-7 extensively overlapped, whereas they colocalized only to a limited number of small puncta in wild type, suggesting that early to late endosome transport is affected (Figure 2U, 2V). Moreover, GFP::RAB-10, which associates with basolateral early endosomes, the Golgi and apical recycling endosomes, displayed a punctate staining pattern in wild type, but formed a few cytoplasmic aggregates as well as appearing on abnormal vacuoles in *chat-1* and *tat-1* mutants (Figure 2G–2I, 2R; Figure S4J–S4L) [38]. In addition, RAB-11, which overlaps with RAB-10 on apical recycling endosomes and the Golgi, also formed aggregates and labeled abnormal vacuoles (Figure 2J–2L, 2S; Figure S4M–S4O) [38]. Finally, we examined the distribution of RME-1 which labels basolateral recycling endosomes [13], [38]. In *tat-1(qx30)* and *chat-1(qx36)* mutants, the distinct punctate staining pattern of RME-1 along basolateral membranes was significantly reduced and RME-1 either became diffuse or formed large aggregates in the cytoplasm, indicating that RME-1-positive recycling endosomes are disrupted (Figure 2M–2O, 2T). The abrogation of endogenous RME-1 pattern was confirmed by the staining of *tat-1* and *chat-1* mutants with anti-RME-1 antibodies (Figure S5D–S5F). RME-1 and RAB-10 localized to distinct endocytic compartments in wild type but partially overlapped on cytosolic aggregated vesicles in *tat-1(qx30)* mutants (Figure 2W, 2X); further suggesting that endocytic trafficking from early to recycling endosomes is defective. Consistent with defective trafficking from early to late endosomes, the number of mature lysosomes strongly labeled by Lysotracker Red significantly decreased in the mutant intestine, whereas ER and Golgi markers appeared normal (Figure S6A–S6J). In epidermis, however, *tat-1(qx30)* and *chat-1(qx36)* mutants accumulated enlarged acidic compartments positive for Lysotracker Red, a phenotype which was previously observed in animals carrying the *tat-1* loss-of-function allele *kr15* (Figure S6K–S6M) [32]. Collectively, our data suggest that endocytic trafficking through early endosomes is severely affected in *tat-1* and *chat-1* mutants, leading to disruption of recycling and late endosomes.

### Cargo recycling and degradation are defective in *chat-1* and *tat-1* mutants

**Figure 3:**
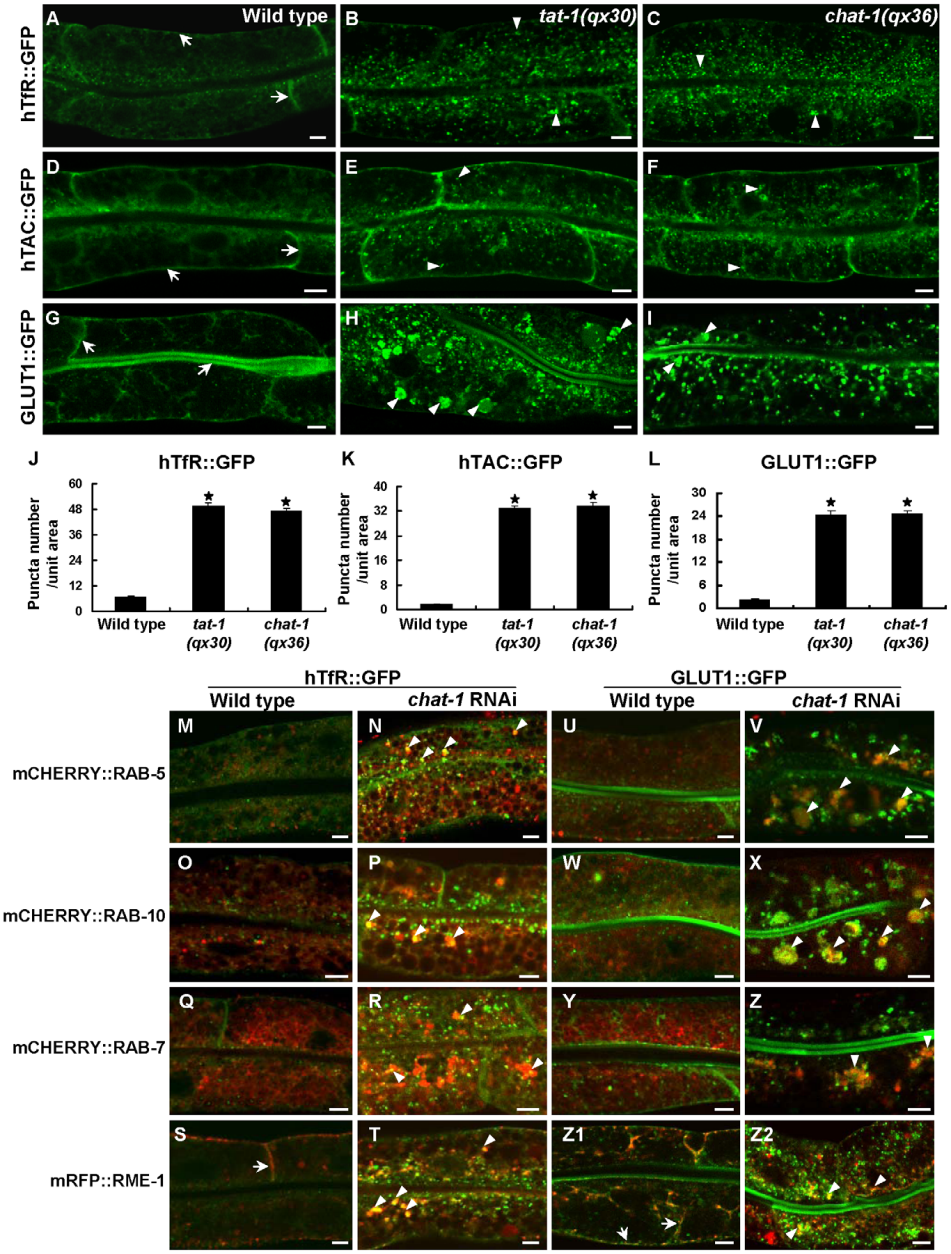
Cargo recycling is disrupted in *tat-1* and *chat-1* mutants. (A–I) Confocal fluorescent images of the intestine in wild type (A, D, G), *tat-1(qx30)* (B, E, H) and *chat-1(qx36)* (C, F, I) that express hTfR::GFP (A–C), hTAC::GFP (D–F) or GLUT1::GFP (G–I). GFP signal was mainly seen on plasma membranes in wild type (arrows) but accumulated intracellularly in *tat-1* and *chat-1* mutants (arrowheads). (J–L) Quantification of intracellular accumulation of hTfR::GFP (J), hTAC::GFP (K) and GLUT1::GFP as shown in (A–I). Data are shown as mean numbers of labeled structures ± SEM. *P<9.0×10^−29^. (M-Z2) hTfR::GFP (M–T) and GLUT1::GFP (U-Z2) are trapped in abnormal early endosomes in *chat-1(RNAi)* animals. Merged images of hTfR::GFP or GLUT1::GFP with mCHERRY RAB-5 (M, N, U, V), mCHERRY::RAB-10 (O, P, W, X), mCHERRY::RAB-7 (Q, R, Y, Z) or mRFP::RME-1 (S, T, Z1, Z2) in wild-type and *chat-1(RNAi)* intestines are shown. Overlap of hTfR or GLUT1 with different endocytic markers is indicated by arrows in wild type and arrowheads in *chat-1(RNAi)* animals. Scale bars: 5 µm.

Because early and recycling endosomes are affected in *tat-1* and *chat-1* mutants, we next investigated whether endocytic recycling is defective by examining the trafficking of hTfR, the human transferrin receptor (hTfR::GFP), and hTAC, the α-chain of the human IL-2 receptor TAC (hTAC::GFP), both of which are recycled in a RAB-10- and RME-1-dependent manner in the *C. elegans* intestine [38]. hTfR::GFP accumulated significantly in the cytosol in *tat-1* and *chat-1* mutants whereas it mainly localized to basolateral membranes in wild type (Figure 3A–3C, 3J). Similarly, increased accumulation of cytosolic hTAC::GFP was also observed in these mutants, albeit to a lesser extent than hTfR (Figure 3D–3F, 3K). These results suggest that recycling of hTfR and hTAC is compromised. Moreover, the glucose transporter 1 (GLUT1), which enters mammalian cells through clathrin-independent endocytosis and constitutively recycles via the Arf6 pathway that require the function of Rab GTPases and RME-1/EHD1 family proteins, primarily localized to apical and basolateral cell membranes when expressed in the *C. elegans* intestine (Figure 3G) [39]. In *tat-1(qx30)* and *chat-1(qx36)* mutants, however, cytosolic accumulation of GLUT1::GFP dramatically increased and some of the signal appeared in abnormal vacuoles, indicating that trafficking of GLUT1 to the plasma membrane was disrupted (Figure 3H, 3I, 3L). Intracellularly accumulated hTfR and GLUT1 were found on vesicles positive for RAB-5, RAB-10 or RAB-7 in *chat-1(RNAi)* animals, especially on vesicles that clustered together, suggesting that they may be trapped in abnormal early endosomes (Figure 3M–3R, 3U–3Z). Consistent with this notion, hTfR and GLUT1 colocalized with RME-1 on basal and lateral membranes in wild type, but partially overlapped with cytoplasmic RME-1 aggregates in *chat-1(RNAi)* animals (Figure 3S, 3T, 3Z1, and 3Z2). hTfR or GLUT1 did not significantly overlap with either CHC-1, a marker for clathrin-coated pits, or Lysotracker Red in wild type or *chat-1(RNAi)* animals (data not shown). These data indicate that loss of *tat-1* and *chat-1* function results in defective recycling of cargoes, which are mainly trapped within abnormal early endosomes.

**Figure 4:**
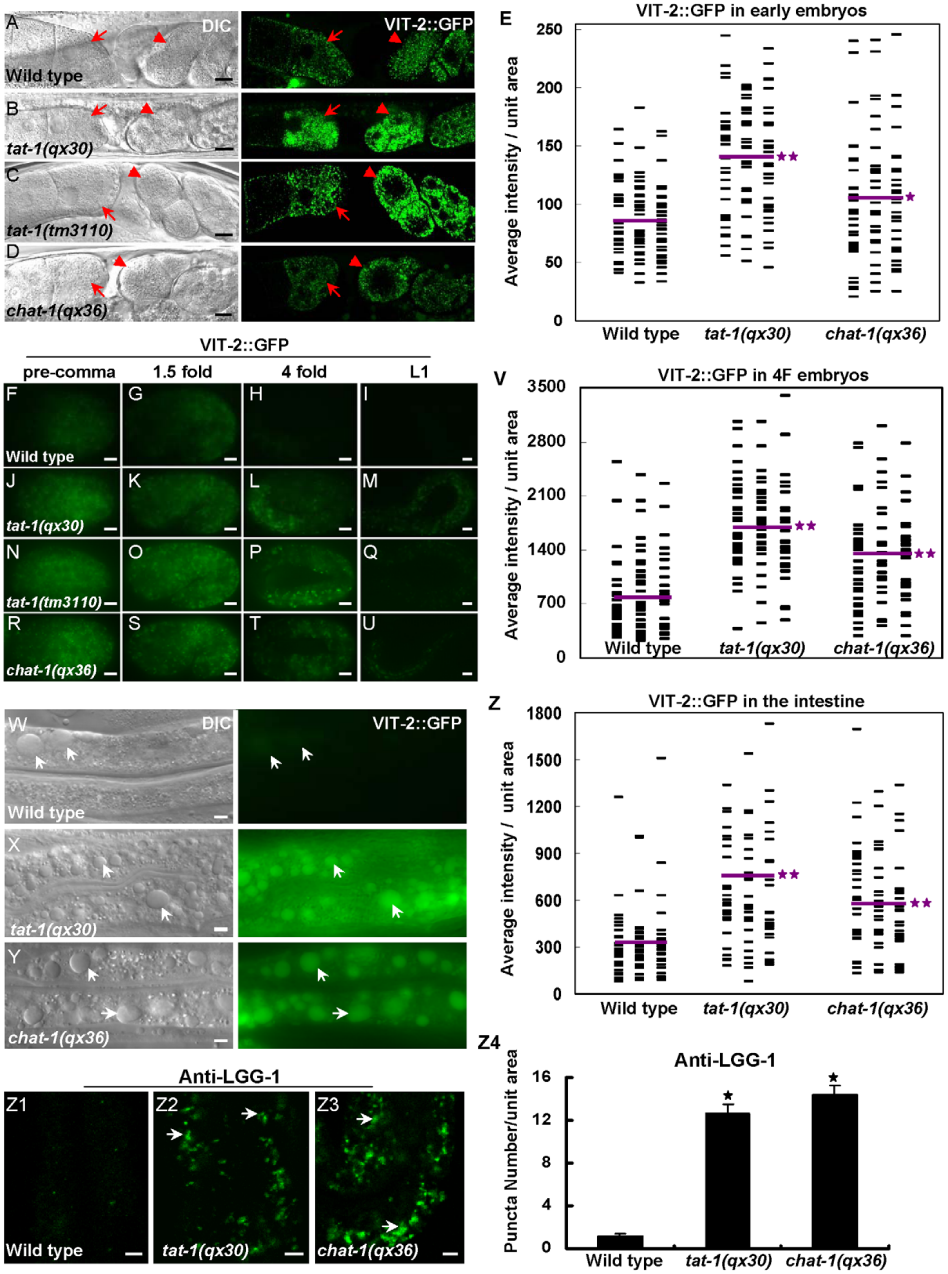
Yolk redistribution and degradation are defective in *tat-1* and *chat-1* mutants. (A–D) *tat-1* and *chat-1* mutations do not affect yolk uptake in oocytes. DIC and confocal fluorescent images of wild type (A), *tat-1(qx30)* (B), *tat-1(tm3110)* (C) *chat-1(qx36)* (D) carrying VIT-2::GFP are shown. Arrows and arrowheads show yolk accumulation in mature oocytes and fertilized embryos, respectively. (F–U) Yolk degradation is affected in *tat-1* and *chat-1* mutants. Fluorescent images of wild-type (F–I), *tat-1(qx30)* (J–M), *tat-1(tm3110)* (N–Q) and *chat-1(qx36)* (R–U) embryos expressing VIT-2::GFP are shown. (W–Y) *tat-1* and *chat-1* mutants accumulate enlarged yolk granules in the intestine. DIC and fluorescent images of wild-type (W), *tat-1(qx30)* (X) and *chat-1(qx36)* (Y) intestine expressing VIT-2::GFP are shown. Enlarged yolk granules are indicated by arrows. (E, V, Z) Quantification of the average total intensity of VIT-2::GFP per unit area in fertilized early embryos (E), 4-fold stage embryos (V) and intestines (Z). The distribution of average total intensity in 96 (E, V) or 84 (Z) unit areas in each genotype is shown. Purple lines represent the average intensity of VIT-2::GFP in each strain. **P<1.8×10^−7^, *P<0.05. (Z1–Z3) *tat-1* and *chat-1* mutants accumulate LGG-1-positive structures in the intestine. DIC and fluorescent images of the intestine in wild type (Z1), *tat-1(qx30)* (Z2), and c*hat-1(qx36)* (Z3) stained with anti-LGG-1 antibodies are shown. Arrows show LGG-1-positive structures. Average number of LGG-1 puncta in each strain was quantified in Z4. Data are shown as mean±SEM. *P<4.0×10^−20^. Scale bars: 5 µm.

As abnormal RAB-7 distribution and fewer mature lysosomes were observed in *tat-1* and *chat-1* intestines, we examined whether the degradative pathway is also affected by using the VIT-2::GFP reporter to monitor yolk trafficking and accumulation [40], [41]. The initial uptake of yolk in both mature oocytes and fertilized embryos was normal in *tat-1* and *chat-1* mutants with no obvious accumulation of VIT-2::GFP in the body cavity (Figure 4A–4E). However, the redistribution of yolk to the gut primordium and their degradation appeared to be affected as significantly more VIT-2::GFP was observed in both early and late embryos as well as L1 larvae in *tat-1* and *chat-1* mutants than in wild type (Figure 4E–4V; Figure S5G–S5I). Moreover, we found that aged *tat-1* and *chat-1* mutants but not wild-type animals (60 h post L4/adult molt), accumulated a large number of big yolk granules in the intestine (Figure 4W–4Z; Figure S5J–S5L). As *tat-1* and *chat-1* adults aged for a shorter period of time (12, 24 or 48 h post L4/adult molt) contained a similar level of yolk in the intestine as in wild type, our data are consistent with compromised yolk degradation in these mutants (data not shown). In addition, *tat-1* and *chat-1* intestines accumulated many LGG-1-postive structures, which were disrupted in animals lacking *atg-3*, *atg-5* or *atg-7*, suggesting that the degradation of autophagic cargo may also be affected (Figure 4Z1–Z4; data not shown) [42].

### CHAT-1 and TAT-1 associate with tubular membranes in sorting and recycling compartments

**Figure 5:**
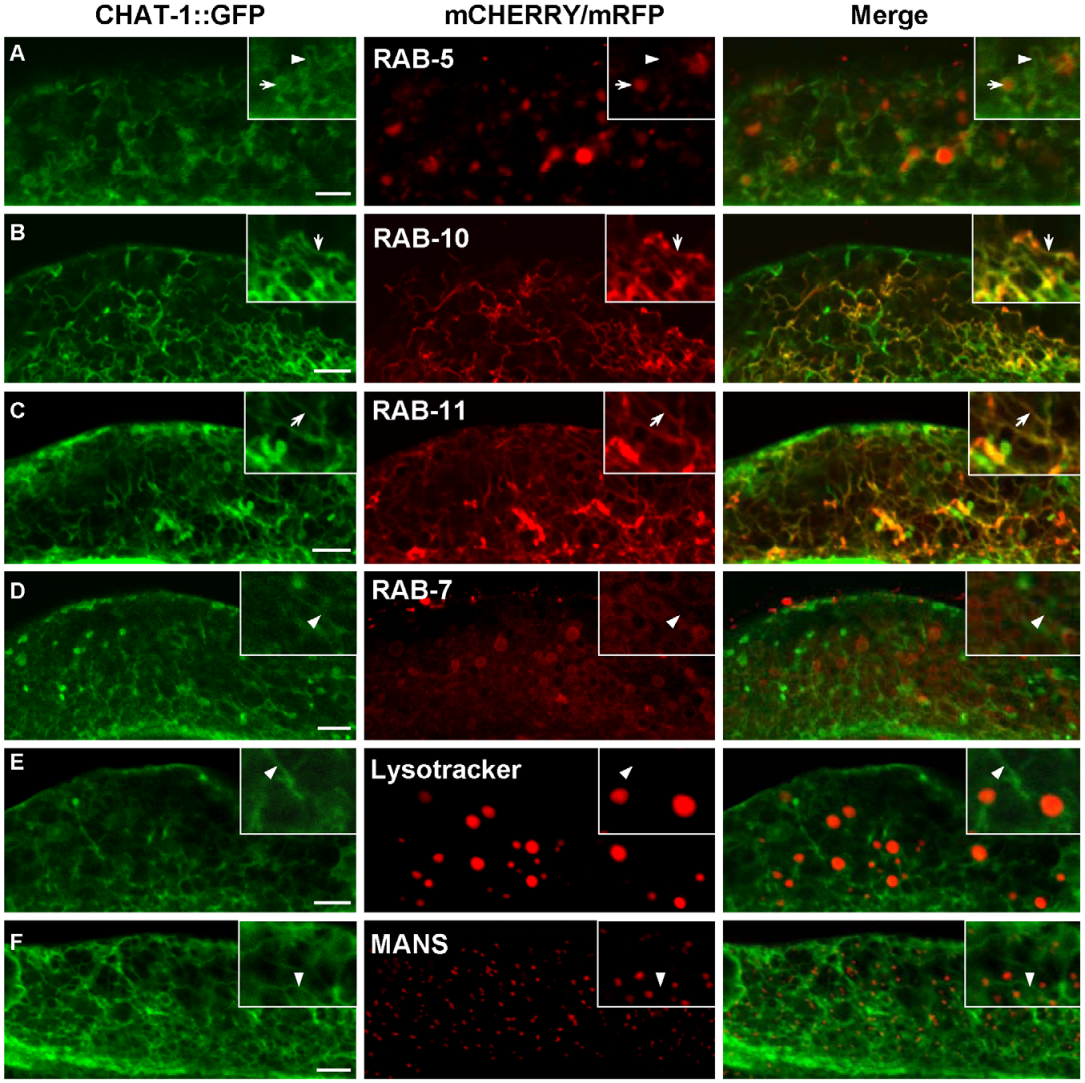
CHAT-1::GFP labels tubular structures in the intestine. CHAT-1::GFP were coexpressed together with mCHERRY or mRFP fusions of various endolysosomal markers in the wild-type intestine. Arrows indicate colocalization of CHAT-1::GFP with different endocytic markers. Arrowheads show tubular structures labeled only by CHAT-1::GFP. Insets show an amplified view with a magnification of *X 1.6*. Scale bars: 5 µm.

To understand how disruption of CHAT-1 and TAT-1 function results in endocytic defects, we examined their subcellular localization in the intestine by coexpressing CHAT-1::CFP and TAT-1::YFP under control of the intestine-specific promoter *vha-6* (P*_vha-6_chat-1::cfp* +P*_vha-6_tat-1::yfp)*. These reporters fully rescued the vacuolation phenotype in *tat-1* and *chat-1* mutants (Figure S2A). TAT-1 and CHAT-1 colocalized to both plasma membranes and intracellular tubular and vesicular structures (Figure 1L; Figure S3A). The intestinal tubular and vesicular localization pattern was also observed when the expression of TAT-1 or CHAT-1 was controlled by the endogenous promoter (Figure S7A, S7B; data not shown). To determine the identities of the cytosolic compartments labeled by CHAT-1 and TAT-1, we coexpressed mCHERRY fusions of different endocytic markers together with CHAT-1::GFP and TAT-1 (P*_vha-6_chat-1::gfp* +P*_vha-6_tat-1*). TAT-1 was included to ensure efficient ER export of CHAT-1::GFP; this combination is subsequently referred to as CHAT-1::GFP for simplicity. CHAT-1::GFP displayed a tubular and vesicular staining pattern, which did not overlap with either the Golgi marker MANS or the lysosomal marker Lysotracker Red, indicating that CHAT-1 is not on the Golgi or mature lysosomes (Figure 5E, 5F). No CHAT-1::GFP was found on the RAB-7-positive ring-like structures, suggesting that it is not enriched on late endosomes or early lysosomes (Figure 5D). However, CHAT-1 partially overlapped with RAB-5 on punctate structures, but not on tubule-like structures that were negative for mCHERRY::RAB-5 (Figure 5A). Thus, a proportion of CHAT-1/TAT-1 may localize to RAB-5-positive early endosomes. We next examined colocalization of CHAT-1 and RAB-10, which associates with endosomes and Golgi compartments [38]. Interestingly, when coexpressed with CHAT-1::GFP, RAB-10 displayed a different staining pattern and mainly localized to CHAT-1-positive tubular structures instead of labeling small cytoplasmic puncta (compare Figure 2G and Figure 5B). Similarly, RAB-11 labeled punctate structures when expressed alone, but colocalized with CHAT-1 on abundant tubules when the two were coexpressed (compare Figure 2J and Figure 5C). Moreover, in animals expressing both mCHERRY::RAB-10 (or RAB-11) and CHAT-1::GFP, the tubular structures became more evident and extensive than in animals carrying only CHAT-1::GFP or animals coexpressing CHAT-1 and RAB-5 or RAB-7 (compare B and C with other panels of Figure 5). These data suggest that RAB-10 and RAB-11 may act together with CHAT-1/TAT-1 to promote extension of the tubular structures.

**Figure 6:**
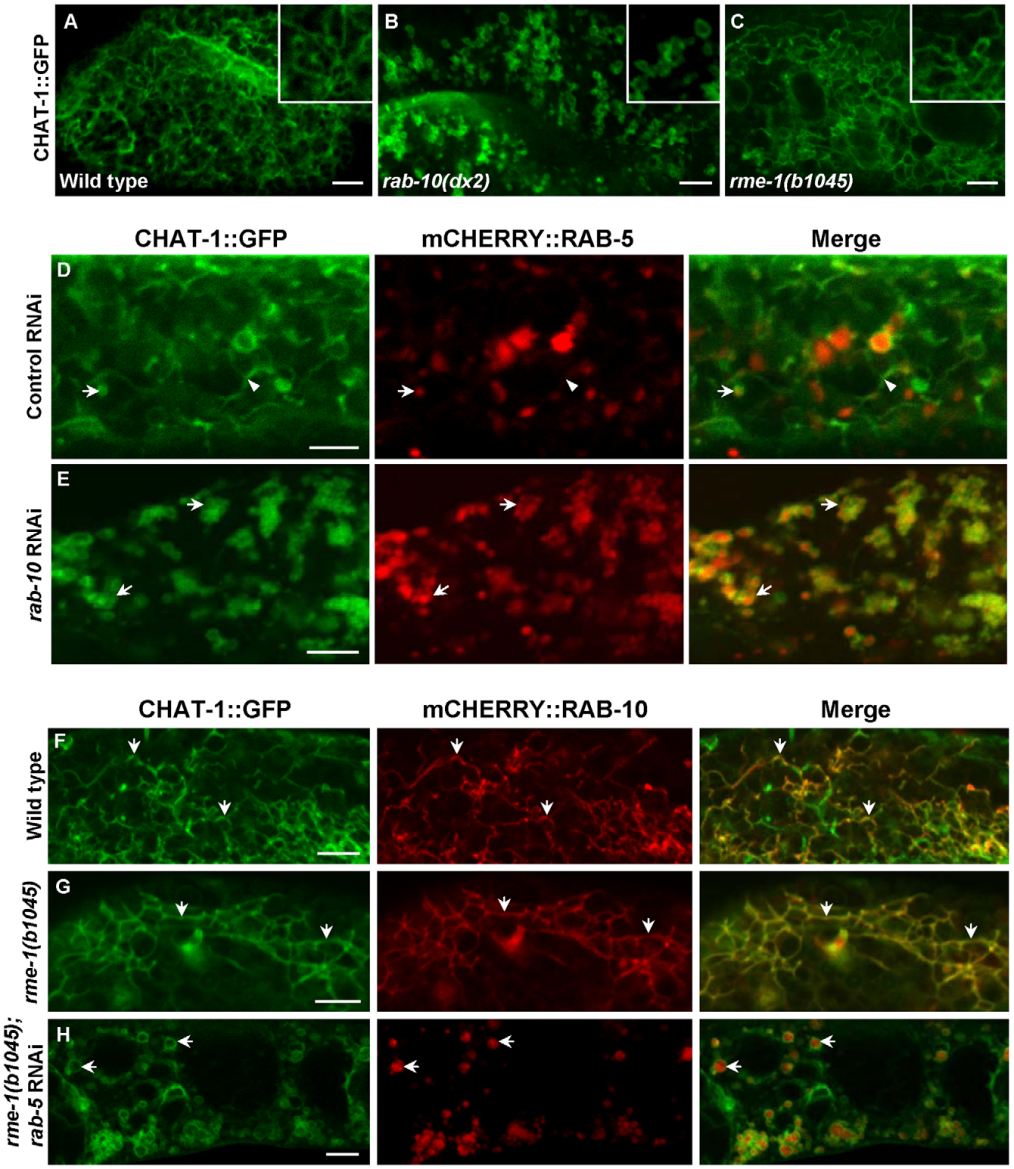
CHAT-1 tubules are disrupted in *rab-10(lf)* but are further extended in *rme-1(lf)* mutants. (A–C) Confocal fluorescent images of CHAT-1::GFP in the intestine of wild type (A), *rab-10(dx2)* (B) and *rme-1(b1045)* (C). Insets have a magnification of *X 1.5*. (D and E) Confocal fluorescent images of CHAT-1::GFP and mCHERRY::RAB-5 in animals treated with control (D) or *rab-10* RNAi (E). Arrows show RAB-5-positive early endosomes and arrowheads indicate CHAT-1-positive tubules. (F–H) Confocal fluorescent images of wild-type (F), *rme-1(b1045)* (G), *rme-1(b1045);rab-5* RNAi (H) intestine expressing CHAT-1::GFP and mCHERRY::RAB-10. Tubular structures labeled by CHAT-1 and RAB-10 (arrows) became further extended in *rme-1(b0145)* mutants (G), but were abolished by *rab-5* RNAi (H). Scale bars: 5 µm.

**Figure 7:**
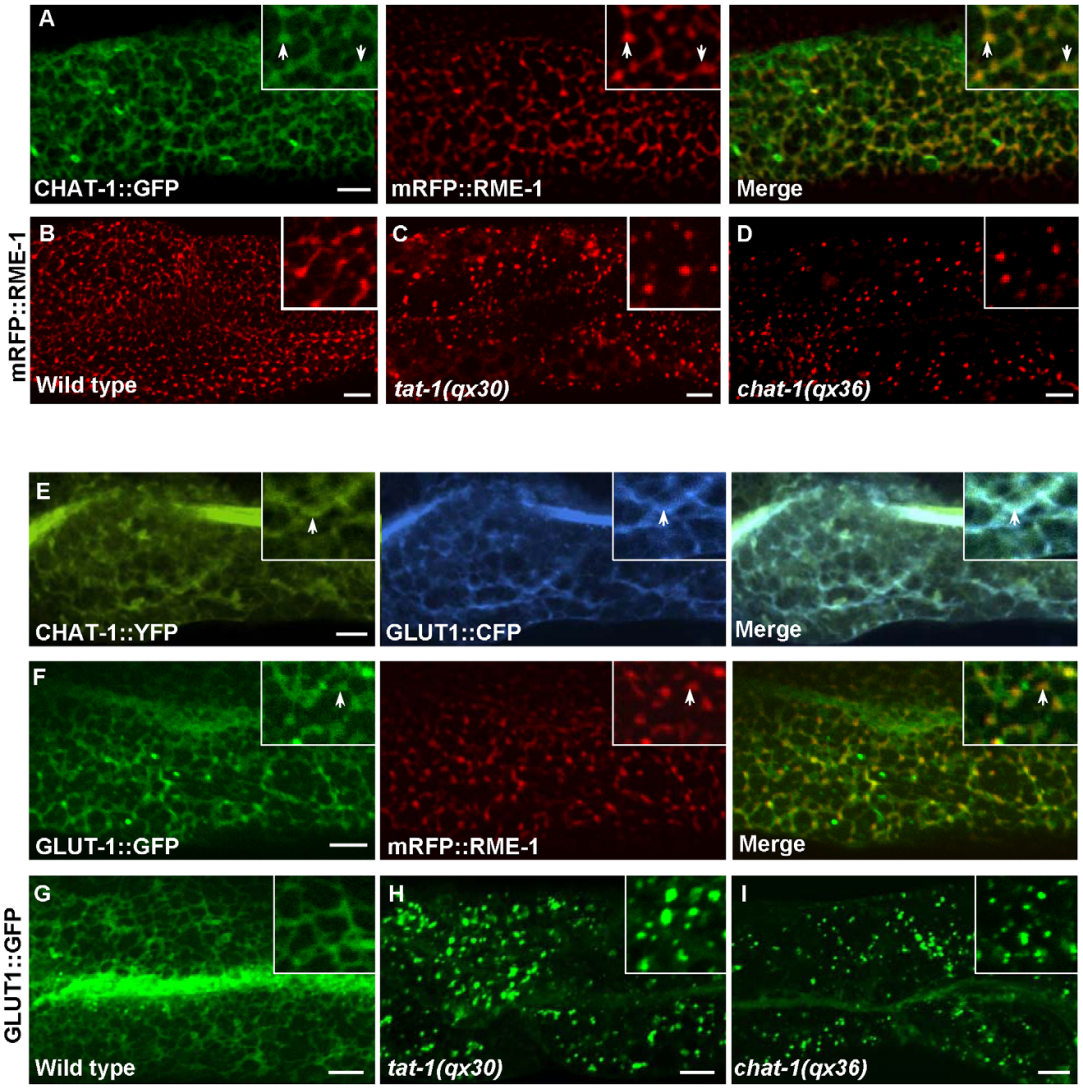
Tubular extensions of sorting and recycling compartments are disrupted in *tat-1* and *chat-1* mutants. (A) Confocal fluorescent images of wild-type intestine expressing both CHAT-1::GFP and mRFP::RME-1. Arrows indicate colocalization of CHAT-1 and RME-1. (B-D) Confocal fluorescent images of mRFP::RME-1 in wild-type (B), *tat-1(qx30)* (C) and *chat-1(qx36)* (D) intestines. RME-1-positive compartments are greatly reduced in number and become globular in *tat-1* and *chat-1* mutants. (E and F) Confocal fluorescent images of wild-type intestine expressing both CHAT-1::YFP and GLUT1::CFP (E) or GLUT1::GFP and mRFP::RME-1 (F). GLUT1 overlaps with CHAT-1 (E) or RME-1 (F) on tubular or tubulo-vesicular structures in the basolateral membrane area (arrows). (G–I) In wild-type intestine, GLUT1::GFP labels tubular structures (G), which are disrupted in *tat-1(qx30)* (H) or *chat-1(qx36)* (I) mutants. Insets have a magnification of *X 1.6* in (A, E and F) and *X 2* in (B–D and G–I). In all panels, images were taken at the top focus plane, which gives a better view of the basolateral membrane area. Scale bars: 5 µm.

As CHAT-1 tubules were labeled by RAB-10 and RAB-11, but not RAB-5 or RAB-7, we reasoned that they may be tubular early endosomes and/or endocytic recycling compartments (ERCs). We first examined the tubular structures in *rab-10(lf)* mutants, in which endocytic transport from early to recycling endosomes is disrupted, and found that CHAT-1 tubules were totally abolished; instead, CHAT-1::GFP overlapped completely with RAB-5 on enlarged early endosomes (Figure 6B, 6E). Inactivation of *rab-10* also disrupted tubules labeled by CHAT-1 and RAB-11 (Figure S7E). Next, we examined the CHAT-1 tubules in *rme-1(lf)* mutants, in which trafficking from recycling endosomes to the plasma membrane is affected, and found that the tubules were not disrupted (Figure 6C). Instead, CHAT-1- and RAB-10- (or RAB-11) positive tubular structures became even more extended when *rme-1* function was lost (Figure 6G; Figure S7D). Conversely, loss of *rab-5* activity completely abrogated the tubular structures in either wild type or *rme-1(b1045)* mutants (Figure 6H; data not shown). RAB-5 and RAB-10 are required in early endosomes and for trafficking from early to recycling endosomes, while RME-1 acts downstream of them to promote membrane fission for releasing recycling carriers [14], [38]. Our data are consistent with the idea that CHAT-1/TAT-1 associates with the tubular membrane of early endosomes and ERCs. Finally, we examined the co-localization of CHAT-1 and RME-1, which is enriched on recycling endosomes [13]. We observed that CHAT-1::GFP overlapped with mRFP::RME-1 on basolateral tubulo-vesicular structures, indicating that CHAT-1 also localizes to RME-1-positive recycling endosomes (Figure 7A).

### CHAT-1 and TAT-1 are required for tubule formation

The formation of tubular extensions in early endosomes, ERCs and recycling endosomes is crucial for sorting and transporting recycling cargoes. We observed that the recycling cargo GLUT1 labeled CHAT-1-positive tubules near basolateral membranes and in the cytoplasm, supporting a role for these tubules in sorting and recycling (Figure 7E; data not shown). GLUT1::GFP was also found on RME-1-positive tubulo-vesicular recycling endosomes (Figure 7F). The tubular membrane structures containing GLUT1::GFP were completely disrupted in *tat-1* and *chat-1* mutants, in which GFP stained cytoplasmic punctate structures (Figure 7G–7I). Moreover, in *tat-1* and *chat-1* mutants, the remaining basolateral RME-1-positive puncta completely lost their tubulo-vesicular morphology and became globular (Figure 7B–7D). These data indicate that TAT-1 and CHAT-1 are required for forming and/or maintaining the tubular extensions of sorting and recycling compartments.

### PS asymmetry across endomembranes is disrupted in *tat-1* and *chat-1* mutants

**Figure 8:**
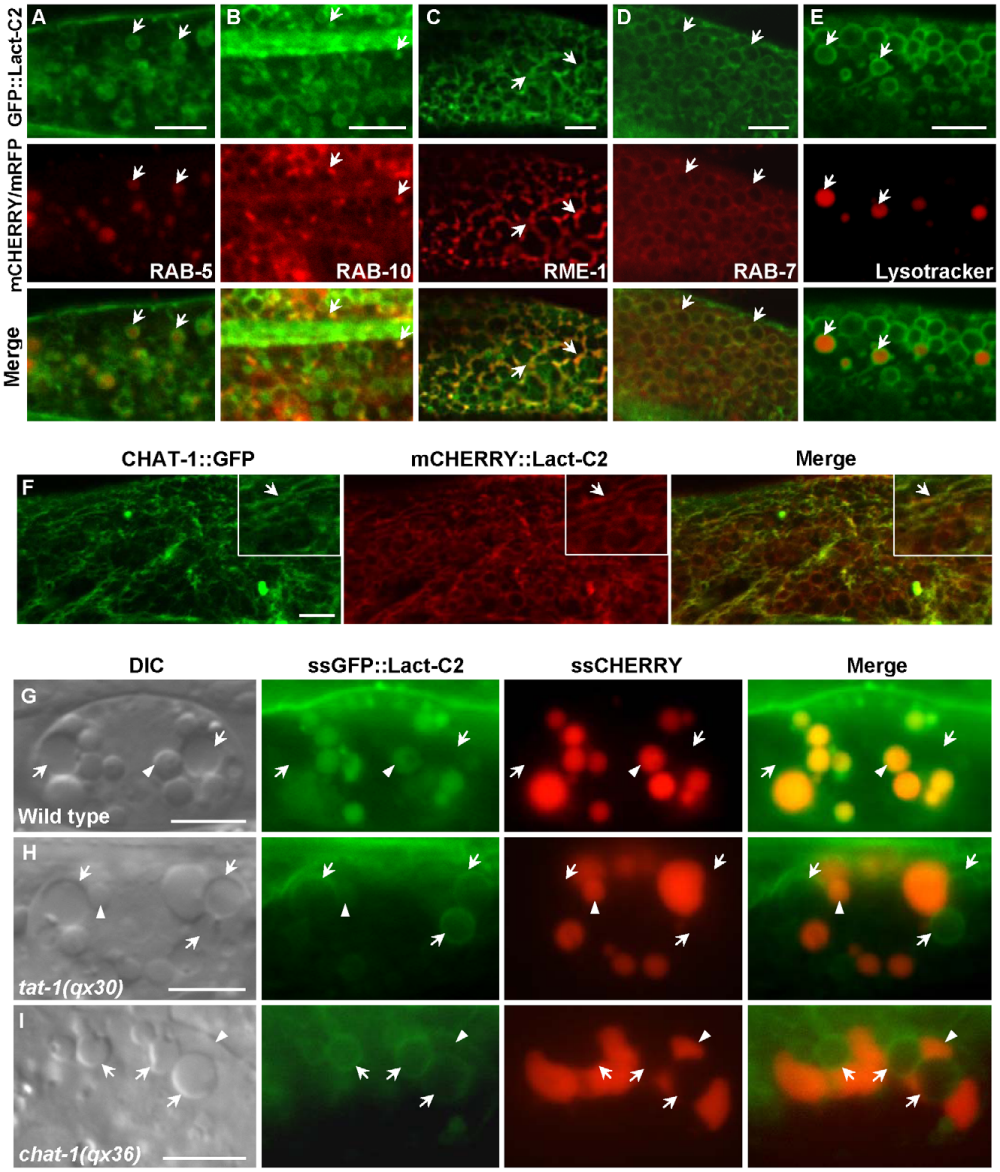
Endomembrane PS asymmetry is disrupted in *tat-1* and *chat-1* mutants. (A–E) PS appears on the cytosolic surface of endocytic compartments. Confocal fluorescent images of wild type carrying GFP::Lact-C2 expressed specifically in the intestine (P*_ges-1_*GFP::Lact-C2) and mCHERRY fusions of RAB-5 (A), RAB-10 (B), RME-1 (C), RAB-7 (D) or stained with Lysotracker Red (E) are shown. Arrows show colocalization of GFP::Lact-C2 and endocytic markers. (F) CHAT-1 tubules are coated by PS. Confocal fluorescent images of wild-type intestine expressing both CHAT-1::GFP and mCHERRY::Lact-C2. CHAT-1 and Lact-C2 coincide on tubular membranes (arrows). Insets show an amplified view with a magnification of *X 1.6*. (G–I) DIC and fluorescent images of coelomocytes that take up and transport ssGFP::Lact-C2 and ssCHERRY endocytosed from the body cavity in wild type (G), *tat-1(qx30)* (H) and *chat-1(qx36)* (I). Arrows show endosomes; arrowheads show lysosomes. Internalized GFP::Lact-C2 is absent from endosomes in wild type but labels endomembanes in *tat-1(qx30)* and *chat-1(qx36)* mutants. Scale bars: 5 µm.

To investigate whether TAT-1 and CHAT-1 regulate endocytic sorting and recycling by maintaining membrane PS asymmetry, we examined PS asymmetry across endomembranes. We first determined PS distribution by expressing the biosensor GFP::Lact-C2 specifically in intestine cells (P*_ges-1_*GFP::Lact-C2) [43]. The cell membranes and surfaces of virtually all internal vesicles were labeled, indicating that PS was exposed on the cytosolic surface of both plasma membranes and various intracellular compartments (Figure S8A). We next coexpressed GFP::Lact-C2 with different endolysosomal markers and found that it labeled intracellular structures that were positive for RAB-5, RAB-7, RAB-10, RME-1 or Lysotracker Red (Figure 8A–8E). Thus, PS appeared on the cytosolic surfaces of recycling, early and late endosomes as well as lysosomes. mCHERRY::Lact-C2 coincided well with CHAT-1::GFP on tubular membranes, indicating that the tubules are coated by PS (Figure 8F).

In coelomocytes, which are scavenger cells that actively endocytose and degrade soluble material, GFP::Lact-C2 (P*_unc-122_*GFP::Lact-C2) stained plasma membranes and the surfaces of endosomes and lysosomes, as observed in intestine cells (Figure S8D, S8E). To determine whether PS is absent from the luminal leaflet of endomembranes in wild type and whether PS asymmetry is affected in *tat-1* and *chat-1* mutants, we focused on coelomocytes, which contain abundant endocytic vesicles that are larger than those in intestine cells. To detect luminal PS in endocytic vesicles, we examined expression of ssGFP::Lact-C2 driven by the *myo-3* promoter (P*_myo-3_*ssGFP::Lact-C2), which after secretion from body wall muscle cells is taken up by coelomocytes through endocytosis and transferred to lysosomes via endocytic transport [44]. In wild-type coelomocytes, the endocytosed GFP::Lact-C2 mainly accumulated in lysosomes as indicated by an endocytic cargo ssCHERRY, whereas no clear GFP signal was detected in endosomes, suggesting that PS is likely absent from the luminal side of endomembranes (Figure 8G; Figure S8F, S8G). Therefore, like the plasma membrane, PS is preferentially distributed on the cytosolic side of endomembranes. Remarkably, we found that in both *tat-1(qx30)* and *chat-1(qx36)* mutants, the internalized GFP::Lact-C2 labeled endosome membranes with a ring-like staining pattern, indicating that PS appeared on the luminal side of endomembranes (Figure 8H, 8I). By contrast, expression of GFP::Lact-C2(AAA), which is deficient in PS binding, gave no or very faint and diffuse GFP signal in endosomes (Figure S8H–S8J). These data suggest that in *tat-1* and *chat-1* mutants, PS asymmetry across endomembranes is disrupted, causing PS to appear on both cytosolic and luminal leaflets of the membrane.

## Discussion

### CHAT-1 acts together with TAT-1 to regulate PS asymmetry and endocytic transport

From a genetic screen for altered distribution of a PS-binding protein, we recovered mutant alleles of *chat-1* and *tat-1*, which displayed identical PS asymmetry phenotypes on both plasma membranes and endomembranes. Mutants of *chat-1* and *tat-1* also showed identical endocytic defects. CHAT-1 and TAT-1 are expressed in the same tissues and colocalize to both plasma membranes and various intracellular compartments in the intestine. Furthermore, CHAT-1 and TAT-1 are co-dependent for exiting the ER, similar to P4-ATPase and Cdc50p proteins in yeast and mammalian cells [28], [36]. Therefore, like Drs2p and Cdc50p, TAT-1 and CHAT-1 may act as a complex to regulate membrane PS asymmetry and endocytic traffic.

### TAT-1/CHAT-1 regulates endocytic sorting

We observed pleiotropic phenotypes associated with the presence of multiple endocytic vesicles in *tat-1* and *chat-1* mutants, many of which can be attributed to defective endocytic sorting. For example, in *tat-1* and *chat-1* mutants, enlarged early endosomes accumulated while recycling and late endosomes were disrupted. The abnormal vacuoles appear to be heterogeneous, since they were labeled by markers of early, late and recycling endosomes as well as early lysosomes [32]. Cargo recycling and degradation are also defective in these mutants. Therefore, loss of *tat-1* and *chat-1* function likely disrupts endocytic sorting through early endosomes, thereby affecting subsequent trafficking through both recycling and degradative pathways.

In a recent study, TAT-1 was found to be required at an early step of endocytosis [32]. Consistent with this, we observed a defect in endocytosis of fluid cargo from both basolateral and apical intestinal cell membranes in *tat-1* and *chat-1* mutants (data not shown). However, defective endocytosis was not seen in oocytes when yolk was taken up. Instead, we observed defects in yolk redistribution and digestion in *tat-1* and *chat-1* embryos (Figure 4).

### TAT-1/CHAT-1 may regulate membrane tubulation by maintaining PS asymmetry

How is endocytic sorting through early endosomes regulated by the P4-ATPase TAT-1 and its chaperone CHAT-1? The tubular elements of early endosomes serve as sorting platforms to enrich and transport transmembrane cargoes. Several lines of evidence indicate that TAT-1 and CHAT-1 are required for generating and/or maintaining the tubular membrane structure. Firstly, *tat-1* and *chat-1* mutants disrupt endocytic transport through early endosomes, leading to the accumulation of enlarged early endosomes positive for RAB-5, which labels the vesicular but not the tubular element. Secondly, TAT-1 and CHAT-1 associate with tubular membranes at sorting and recycling compartments, which contain the recycling cargo GLUT1. Thirdly, loss of *tat-1* and *chat-1* function abolishes the tubular membrane structure containing GLUT1 and disrupts the tubulo-vesicular morphology of RME-1-positive recycling endosomes.

**Figure 9:**
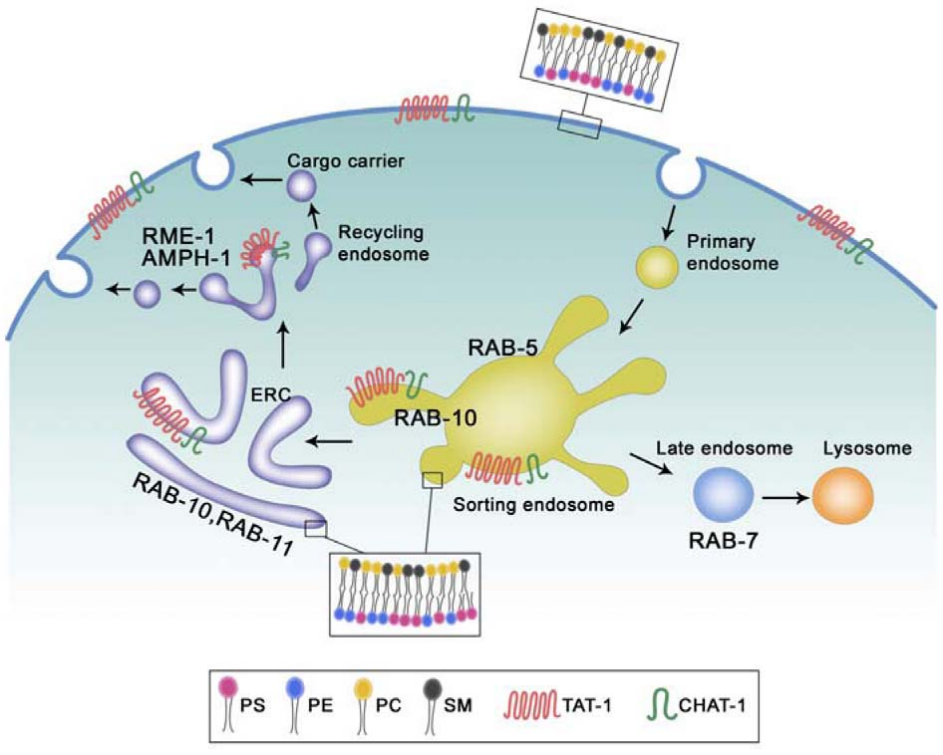
Proposed functions of TAT-1/CHAT-1 in endocytic sorting and recycling. TAT-1 and CHAT-1 localize to plasma membranes and to tubular early endosomes, ERCs and recycling endosomes where they regulate sorting and recycling events by restricting PS to the cytosolic membrane leaflet. TAT-1/CHAT-1 probably promotes tubule formation by enriching PS on the cytosolic leaflet, thereby affecting membrane curvature. RAB-10 and RAB-11 may also contribute to membrane tubulation in early endosomes and ERCs, whereas RME-1/AMPH-1 is involved in membrane fission and tubulation of recycling endosomes. PS, phosphatidylserine; PE, phosphatidylethanolamine; PC, phosphatidylcholine; SM, sphingomyelin.

Phospholipids can have significant effects on membrane curvature when their distributions between the two membrane leaflets are altered. It was observed that addition of exogenous phospholipids including PS to the outer leaflet of discoid platelets caused expansion of the outer surface in the form of numerous extensions [45]. In another case, incorporation of PS, phosphatidylethanolamine and phosphatidylcholine into the outer leaflet of discoid erythrocytes increased the outer membrane surface area and induced a crenated shape with a higher ratio of external to internal surface area, whereas transverse diffusion of exogenous phospholipids from the outer to the inner leaflet reversed the shape change [17], [45]. Our findings that CHAT-1-assoicated tubules are coated by PS and that loss of *tat-1* and *chat-1* function disrupts PS asymmetry of endomembranes and abrogates tubular extensions strongly suggest a role of PS and/or PS asymmetry in membrane tubulation. As an aminophospholipid transporter, TAT-1/CHAT-1 may catalyze the active translocation of PS from the luminal to the cytosolic leaflet of endomembranes, which results in a high ratio of PS in the cytosolic leaflet versus the inner leaflet and an increased outer monolayer area, leading to the deformation of membranes into tubular extensions [46] (Figure 9). Consistent with this, we observed extensive tubular membrane structures labeled by the PS biosensor mCHERRY::Lact-C2 in animals overexpressing CHAT-1 and TAT-1.

Our data suggest that RAB-10 and RAB-11 may also contribute to tubule formation or extension as both of them, when overexpressed, become enriched on CHAT-1::GFP-positive tubules and enhance tubule extension, whereas loss of *rab-10* function completely disrupts tubular structures and traps CHAT-1 on enlarged RAB-5-positive early endosomes (Figure 9). However, we do not know whether RAB-10 plays a direct or indirect role in this process, or how its function is achieved. One interesting possibility is that RAB-10 and RAB-11 may contribute to tubule formation by regulating membrane-bending proteins, as BAR proteins are found to interact with a variety of effectors of small GTPases including Rabs [9].

RME-1 and the BAR protein AMPH-1 were recently shown to cooperatively regulate endocytic recycling in *C. elegans* intestine and to tubulate liposomes in vitro [14]. We found that PS-coated RME-1-positive recycling endosomes lost their tubulo-vesicular morphology in *tat-1* and *chat-1* mutants, suggesting that TAT-1/CHAT-1-regulated PS translocation may be involved in membrane tubulation mediated by RME-1/AMPH-1 (Figure 9). In addition, our observation that CHAT-1-RAB-10 (or RAB-11) tubules are further extended in *rme-1(lf)* mutants is consistent with the proposed function of EHD/RME-1 proteins in mediating membrane fission to generate recycling carriers [14], [16].

Our data support a role of TAT-1/CHAT-1 in membrane tubulation for cargo sorting and recycling. However, we do not know how the sorting of cargo for degradation may be affected by loss of *tat-1* and *chat-1* function. Interestingly, it was reported that *tat-1(lf)* mutants accumulate giant multi-vesicular bodies (MVBs) in hypodermal cells [32]. As translocation of PS and PE across the membrane bilayer is thought to provide the driving force for membrane bending [17], [47], [48], we suspect that loss of TAT-1 and CHAT-1 function may lead to defective budding from multi-vesicular compartments of early endosomes, thereby affecting the sorting of cargo into the degradative pathway. Further experiments need to be performed to test this hypothesis, especially the examination of MVBs in intestine cells.

## Materials and Methods

### *C. elegans* strains

Strains of *C. elegans* were cultured and maintained using standard protocols [49]. The N2 Bristol strain was used as the wild-type strain except for polymorphism mapping which used Hawaiian strain CB4856. Mutations used are described in *C. elegans* II [50] unless otherwise indicated. Linkage group I (LGI): *rab-10(dx2)* [38]. LGIII: *dpy-18 (e364am)*, *bli-5(e518)*, *tat-1(qx30)*, *tat-1(qx23)*, *tat-1(qx24)*, *tat-1(tm3110)* (this study). LGIV: *dpy-9(e12)*, *unc-17(e245)*, *dpy-13(e184sd)*, *unc-8(n491sd)*, *chat-1(qx36)*, *chat-1(ok1681)* (this study). LGV: *unc-62(e644)*, *dpy-11(e224)*, *rme-1(b1045)* [13]. LGX: *lon-2(e678)*, *unc-27(e155)*.

*bIs34* (*pcc1*RME-8::GFP) [51], *cdIs73* (*pcc1*RME-8::mRFP) [52] and *cdIs97* (*pcc1*mCHERRY::CUP-5) [53] were kindly provided by Dr. Hanna Fares (University of Arizona, Tucson, AZ). *pwIs216* (P*_vha-6_*mRFP::RME-1), *pwIs112* (P*_vha-6_*hTAC::GFP) [38], and *pwIs717* (P*_vha-6_*hTfR(short)::GFP) [14] were kindly provided by Dr. Barth Grant (Rutgers University, Piscataway, NJ). *yqIs25* (P*_lgg-1_*LGG-1::GFP) was kindly provided by Dr. Chonglin Yang (Institute of Genetics and Developmental Biology, Beijing).

Other strains carrying integrated or transgenic arrays used in this study are as follows:

*pwIs50* (P*_lmp-1_*LMP-1::GFP) [52], *bIs1* (VIT-2::GFP) [40], *qxIs162* (P*_ges-1_*mCHERRY::TRAM), *qxIs110* (P*_ges-1_*mCHERRY::RAB-5), *qxIs111* (P*_ges-1_*mCHERRY::RAB-7), *qxIs213* (P*_ges-1_*mCHERRY::RAB-10), *qxIs195* (P*_ges-1_*GFP::RAB-10), *qxIs154* (P*_unc-122_*GFP::Lact-C2), *qxIs165* (P*_ges-1_*GFP::Lact-C2), *qxIs150* (P*_chat-1_*CHAT-1::CFP), *qxIs188* (P*_tat-1_*TAT-1::YFP +P*_chat-1_*CHAT-1::CFP), *qxIs143* (P*_tat-1_*TAT-1::GFP), *qxEx1753* (P*_vha-6_*CHAT-1::GFP), *qxEx2966* (P*_vha-6_*TAT-1::YFP + P*_vha-6_*CHAT-1::CFP), *qxEx2622* (P*_vha-6_*CHAT-1::GFP + P*_vha-6_*TAT-1), *qxEx1736* (P*_hsp_*ssGFP::Lact-C2), *qxEx1999* (P*_hsp_*ssGFP::Lact-C2(AAA)), *qxEx1398* (P*_myo-3_*ssGFP::Lact-C2), *qxEx1533* (P*_myo-3_*ssGFP::Lact-C2(AAA)), *qxEx2841* (P*_vha-6_*GFP::RAB-5), *qxEx2616* (P*_vha-6_*GFP::RAB-7), *qxEx1317* (P*_ges-1_*GFP::RAB-11), *qxEx2247* (P*_vha-6_*GLUT1::GFP), *qxEx2726* (P*_hsp_*ssCHERRY), *qxEx1207*(P*_tat-1_*MANS::mCHERRY), *qxEx1914* (P*_vha-6_*mCHERRY::Lact-C2), *qxEx1867* (P*_vha-6_*mCHERRY::RAB-11), *qxEx3265* (P*_vha-6_*GLUT1::CFP + P*_vha-6_*CHAT-1::YFP + P*_vha-6_*TAT-1).

### Isolation, mapping, and cloning of *chat-1* and *tat-1*

TTR-52 is a secreted protein that specifically recognizes apoptotic cells through its binding to surface exposed phosphatidylserine (PS) [34]. In wild-type embryos carrying P*_hsp_*TTR-52::mCHERRY, apoptotic cells are surrounded by mCHERRY, which is absent from the surface of living cells. In order to understand how the PS engulfment signal is regulated, we performed a forward genetic screen to look for mutants which disrupt or alter the staining of apoptotic cells by TTR-52::mCHERRY. From this screen, we isolated the *qx36* mutant and several alleles of *tat-1*, which resulted in TTR-52::mCHERRY staining of virtually all cells, both dying and living [34].

*qx36* was mapped to linkage group IV. Two rounds of three-point mapping were performed using *unc-17* (−3.11) *dpy-13* (0.00) and *dpy-13* (0.00) *unc-8* (+3.29), which mapped *qx36* to a small genetic interval between 0.00–1.02. Transformation rescue experiments were performed and one fosmid clone in this region, WRM0637aA04, rescued the *qx36* defect. Long PCR fragments covering different open reading frames within this fosmid were tested and only the fragment covering R08C7.2 possessed rescue activity. R08C7.2 encodes a Cdc50p-like protein of 348 amino acids, which we named *chat-1* (chaperone of *tat-1*). Sequencing of the locus in the *qx36* mutant identified a C to T transition, which resulted in a premature stop codon after Leu 94. Given that similar phenotypes were observed in *ok1681*, a *chat-1* deletion mutant containing an 1152 bp deletion that removes the region from exon 2 to intron 5 of the *chat-1* gene, or when *chat-1* is inactivated by RNAi, *qx36* is probably a null or strong loss-of-function mutation of *chat-1*.

*qx30* was mapped to the right arm of linkage group III (LGIII). Further three-factor mapping and subsequent single nucleotide polymorphism (SNP) mapping were performed using *dpy-18* (8.65) *bli-5* (21.21) and various SNP markers. *qx30* was mapped between genetic map positions 17.26 (Snp-Y49E10(2)) and 17.85 (Snp-Y111B2(9)). Since the *tat-1* gene locates at 17.59 of LGIII, a transformation rescue experiment was performed and a DNA fragment containing the *tat-1* gene fully rescued the *qx30* defect. The sequence of the *tat-1* gene was determined in *qx30* and three other mutants, *qx22*, *qx23*, *qx24*, which are allelic to *qx30*. The *qx22* and *qx23* mutants carry the same mutation that results in substitution of the Ala at codon 627 with Val, whereas the *qx24* mutant has a splicing mutation (G to A transition) at the junction of intron 12 and exon 13. The *qx30* mutant has a G to A transition that results in a premature stop codon after Leu 1028.

### RNAi

To inactivate *tat-1* and *chat-1* by RNAi, dsRNA synthesized in vitro (550 ng/µl) was injected into the gonad of young adult hermaphrodites (see Table S1 for primer sequences). Embryos laid between 16 to 24 h post-injection were either used for analyzing PS asymmetry or cultured until the L4 larval or young adult stage for examining intestinal phenotypes. We found that *tat-1* RNAi significantly diminished the expression of *tat-1*. For example, 65% of embryos (n = 40) transgenic for P*_tat-1_tat-1::gfp* had bright GFP fluorescence before injection, but only 4% of them showed similar GFP intensity after injection. RNAi treatment of *tat-1* also resulted in a 56% reduction of TAT-1::GFP expression in larvae (n = 40). Similarly, 100% of animals carrying an integrated P*_chat-1_chat-1::gfp* reporter had strong GFP fluorescence before injection, but after *chat-1* RNAi treatment, 99% had only weak GFP signal (n = 30). For *rab-5* and *rab-10* RNAi, the bacterial feeding protocol was used as described before [54]. Briefly, L3 larvae were treated with either *rab-5* RNAi (I-4J01) or control RNAi (pPD129.36) and adult animals of the same generation were scored as most F1 progeny die due to inactivation of *rab-5*. For *rab-10* RNAi, L3 or L4 larvae were treated with either *rab-10* RNAi (I-3G23) or control RNAi (pPD129.36) and F1 progeny were examined at the adult stage.

### Examination of PS asymmetry and yolk accumulation

The *ex vivo* staining of dissected gonads by Annexin V was performed as described before [55]. To determine membrane PS asymmetry in embryos, mixed stage animals carrying P*_hsp_*ssGFP::Lact-C2 or P*_hsp_*ssGFP::Lact-C2(AAA) were incubated at 33°C for 1 h followed by recovery at 20°C for 2.5 h before examination. To examine PS asymmetry across endomembranes in coelomocytes, mixed stage P0 animals carrying both P*_myo-3_*ssGFP::Lact-C2 and P*_hsp_*ssCHERRY were incubated at 33°C for 0.5 h to induce the expression and secretion of ssCHERRY. Culture was continued at 20°C for one more generation before F1 adults were examined. This long incubation allows the complete uptake and transport of ssCHERRY to lysosomes, which otherwise is seen in the body cavity and many tissues other than coelomocytes. To examine yolk uptake in oocytes, L4 hermaphrodites from the strains indicated were aged for 12 and 24 h and confocal images were taken with the same exposure time. The uptake of yolk was observed at the same two time points in wild type and *tat-1* and *chat-1* mutants. Images of animals that were aged to 24 h post L4/adult molt are shown in Figure 4. To examine yolk accumulation in the intestine, L4 larvae were aged for 12, 24, 48, and 60 h and images were taken with the same exposure time. No obvious difference in yolk accumulation was observed in wild-type, *tat-1* or *chat-1* intestines when animals were aged less than 60 h.

### Quantification of cargo recycling

Intracellular accumulation of hTfR::GFP, hTAC::GFP and GLUT1::GFP was quantified by determining the number of labeled structures within a 250 µm^2^ area in the intestine. Lysotracker Red-positive granules and mRFP::RME-1-positive vesicles were counted within a unit area of 150 µm^2^. LGG-1 puncta and GFP::RAB-10- or GFP::RAB-11-labeled aggregated structure (clustering of >2 labeled puncta) were scored within a unit area of 500 µm^2^. Five different areas were chosen and quantified in each animal at the L4 larval or young adult stage and 8 animals were scored for each strain. The average total intensity per unit area of GFP::RAB-5 and GFP::RAB-7 in the intestine and VIT-2::GFP in fertilized early embryos was measured using Image J 1.42q software. For GFP::RAB-5 and GFP::RAB-7, 5 different areas (40 µm^2^ each) in the intestine of L4 larvae were chosen for each animal and 8 animals were quantified. For VIT-2::GFP in fertilized early embryos, 3 different regions (20 µm^2^ each) were chosen for each embryo and 32 embryos were scored. Axiovision Rel. 4.7 software (Carl Zeiss, Inc.) was used to quantify average total intensity per unit area of VIT-2::GFP in 4-fold stage embryos or in the intestine of aged adult (60 h post L4/adult molt). 3 different regions (12.6 µm^2^ each) were chosen for each animal and 32 embryos or 28 intestines were quantified. Student's two tailed unpaired *t*-test was performed and the *P* value was indicated in the figure legend.

### Antibody generation and immunostaining

CHAT-1(74-306) or full-length RAB-7 protein tagged with six Histidine residues (CHAT-1(74-306)-His_6_ or RAB-7-His_6_) was expressed in and purified from *E. coli* and used to raise rat polyclonal antibody against CHAT-1 or RAB-7. The antibodies were further purified by incubating 3 ml rat serum with nitrocellulose membrane strips containing 5 mg CHAT-1(74-306)-His_6_ or RAB-7-His_6_ protein. Bound antibodies were eluted with 100 mM glycine-HCl (pH 2.5). Purified anti-RAB-7 antibody recognized a single band of expected size (24 KD) in a western blot analysis using lysate prepared from mixed staged wild-type worms, while purified anti-CHAT-1 antibody failed to detect endogenous CHAT-1 expression in wild-type animals, but recognized CHAT-1::GFP (64 KD) or CHAT-1- His_6_ (39 KD) when the western blot was performed using lysate prepared from transgenic animals expressing CHAT-1::GFP or CHAT-1-His. In a whole-mount immunostaining experiment, anti-CHAT-1 antibody stained plasma membranes in wild-type but not *chat-1(qx36)* embryos or oocytes. RAB-7 antibody staining revealed a specific pattern reminiscent of GFP::RAB-7 in the intestine of wild-type but not *rab-7* RNAi-treated animals except for the staining of apical membranes which appears to be non-specific. Monoclonal anti-GFP antibody was purchased from Roche (USA) and anti-RME-1 antibody was obtained from Developmental Studies Hybridoma Bank (University of Iowa, USA). For immunostaining, mixed stage embryos or dissected intestines were fixed with methanol/acetone followed by blocking in phosphate buffered saline (PBS) containing 1% BSA and 10% fetal calf serum for 2 h at 4°C. The samples were then incubated with primary antibodies in blocking buffer at 4°C overnight with 1:50 dilution for RAB-7, RME-1, CHAT-1 antibodies, 1:200 for GFP antibody and 1:500 for LGG-1 antibody. After washing three times with PBST (PBS + 0.2% Tween 20), the samples were incubated with secondary antibody conjugated to Alexa-488 or Alexa-546 (Molecular Probes) at a 1:50 dilution for 2 h at room temperature. The stained samples were washed three times as before and mounted in 15% VECTASHIELD mounting medium with DAPI (VECTOR) and visualized using a Zeiss LSM 510 Meta inverted confocal microscope.

### Microscopy and imaging analysis

DIC and fluorescent images were captured with a Zeiss Axioimager A1 equipped with epifluorescence and an AxioCam monochrome digital camera and were processed and viewed using Axiovision Rel. 4.7 software (Carl Zeiss, Inc.). A 100x Plan-Neofluar objective (NA1.30) was used with Immersol 518F oil (Carl Zeiss, Inc.). For confocal images, a Zeiss LSM 5 Pascal inverted confocal microscope with 488, 543, 514, 458 and 405 lasers was used and images were processed and viewed using LSM Image Browser software.

### Plasmid construction

The sequences of the PCR primers mentioned in this section are presented in 1. P*_tat-1_tat-1::gfp* was constructed by ligating the *tat-1* minigene (*tat-1A*) containing 2.4 kb of the promoter sequence digested from P*_tat-1_tat-1::flag* to pPD49.26-gfp2 through the Hind III and Nhe I sites [31]. To generate P*_tat-1_tat-1::yfp*, the 2.4 kb promoter sequence of *tat-1* was first amplified using primers PJCY16/17 and cloned into pPD49.26-yfp2 via the Hind III/Xma I sites. The resulting P*_tat-1_yfp* was then ligated with full length *tat-1* cDNA *(tat-1A)* amplified with primers PBHC154/PJCY4 through the Nhe I site. To construct P*_vha-6_tat-1*, the *tat-1* minigene (*tat-1A*) was amplified using primers PBHC123/124 and cloned into P*_vha-6_* through the Kpn I site. P*_vha-6_tat-1::yfp* was generated by fusion PCR with three pairs of primers: (1) PPFG211/PWZ263 for amplifying the *vha-6* promoter, (2) PWZ625/PWZ512 for amplifying sequences of the *tat-1* minigene (*tat-1A*), *yfp* and the 3′UTR of the *unc-54* gene and (3) PWZ624/PPFG199 for the final round of PCR amplification to create P*_vha-6_tat-1::yfp* [56]. To generate P*_chat-1_chat-1a* (P*_chat-1_chat-1*), the 2 kb promoter sequence of *chat-1* was amplified using primers PJCY21/9 and cloned into pPD49.26 through the Sph I/Xma I sites. The resulting P*_chat-1_* was then ligated with either the genomic fragment of *chat-1a* amplified by PJCY18/34 or the 1.1 kb genomic sequence of *chat-1b* amplified by PJCY18/35 through the Nhe I site to generate P*_chat-1_chat-1a* and P*_chat-1_chat-1b*. P*_hsp_chat-1c* was constructed by ligating the 1.2 kb genomic fragment of *chat-1c* amplified using primers PJCY20/PYJ7 to pPD48.78 and pPD49.83 through the Nhe I and Nco I sites. To construct P*_chat-1_chat-1cfp*, the 2 kb promoter sequence of *chat-1* was first cloned into pPD49.26-cfp2 via the Sph I and Xma I sites, which was then ligated with the genomic fragment of *chat-1* amplified with PJCY18/6 through the Nhe I site. The genomic fragment of *chat-1* was similarly cloned into P*_vha-6_*, P*_vha-6_cfp2* and P*_vha-6_gfp2* through the Nhe I site to generate P*_vha-6_chat-1*, P*_vha-6_chat-1::cfp* and P*_vha-6_chat-1::gfp*, respectively. To construct various endocytic markers for specific expression in the intestine, genomic fragments (*rab-5*, *rab-7*, *rab-10*, *rab-11.1*) were PCR-amplified and cloned into P*_vha-6_gfp1*, P*_vha-6_mcherry1*, P*_ges-1_gfp1* or P*_ges-1_mcherry1* through the Kpn I, Kpn I/EcoR V (*mcherry::rab-7*) or Kpn I/Sma I (*mcherry::rab-5*) sites. The sequences of ER (TRAM: translocating chain associating membrane protein) and Golgi (MANS: mannosidase short) reporters were determined as described before [57], and amplified using primers PYJ44/45 and PYJ40/53, respectively. The resulting PCR fragments were then cloned into P*_ges-1_mcherry1* and P*_ges-1_mcherry2* to create P*_ges-1_mcherry::tram* and P*_ges-1_mans::mcherry*, respectively. To generate the GLUT1::GFP reporter, a 2.5 kb genomic fragment of R09B5.11 was amplified with primers PBHC204/205 and cloned into P*_vha-6_gfp2* through the Nhe I site. To generate GFP::Lact-C2 constructs for specific expression in coelomocytes and intestine, a Lact-C2 fragment was amplified from PVM-LACT-1 (Haematologic Technologies Inc.) using primers PBHC144/145 or PBHC147/146 and cloned into P*_unc-122_gfp1* and P*_ges-1_gfp1* through the EcoR V/Sac I and Kpn I sites, respectively. To construct a secreted GFP::Lact-C2, a Lact-C2 fragment was amplified with PBHC146/147 and cloned into P*_hsp_gfp1* or P*_myo-3_gfp1* through the Kpn I site. The resulting P*_hsp_gfp::lact-c2* and P*_myo-3_gfp::lact-c2* were then ligated with a 102 bp fragment containing a synthetic secretion signal amplified from pPD95.85 using primers PYZ203/206 through the Nhe I/Spe I sites to create P*_hsp_ssgfp::lact-c2* and P*_myo-3_ssgfp::lact-c2*. The secretion signal was also cloned into P*_hsp_cherry* to get P*_hsp_sscherry*. To create the secreted Lact-C2(AAA) construct, point mutations (W26A, W33A, F34A) were introduced by site-directed mutagenesis (QuickChange; Stratagene, USA) into PVM-LACT-1 [19], which was further cloned into P*_hsp_gfp1* and P*_myo-3_gfp1* using similar strategies as described above.

## Supporting Information

Figure S1 TAT-1 and CHAT-1 are required for maintaining plasma membrane PS asymmetry. (A)Schematic diagram of the *C. elegans* tat-1 gene. Filled boxes represent exons; thin lines are introns. The arrow shows the direction of transcription. The positions of the four *tat-1* mutations (one deletion and three intragenic mutations) are indicated. (B–G) DIC and confocal fluorescent images of various *tat-1(lf)* and *chat-1(lf)* embryos expressing the biosensor ssGFP::Lact-C2 driven by heat-shock promoters (P*_hsp_*ssGFP::Lact-C2). Arrows indicate apoptotic cells surrounded by ssGFP::Lact-C2. (H-J) DIC and confocal fluorescent images of wild-type (H), *tat-1(qx30)* (I) and *chat-1(qx36)* (J) embryos expressing a secreted biosensor GFP::Lact-C2(AAA) driven by heat-shock promoters. GFP::Lact-C2(AAA) was secreted but failed to label the surfaces of either living or dying cells (indicated by arrows) in wild-type, *tat-1(qx30)* or *chat-1(qx36)* embryos. (K–L) Inactivation of *tat-1* and *chat-1* by RNAi results in abnormal vacuoles in the intestine. DIC images of the intestine of *chat-1(RNAi)* (K) and *tat-1(RNAi)* (L) animals are shown. Arrows indicate abnormal vacuoles. Scale bars: 5 µm.
(1.29 MB PDF)

Figure S2 Molecular cloning of *chat-1*. (A) Cloning of *chat-1*. The top bar indicates the genetic map of the *chat-1* genomic region and the lower panels show the rescue of *chat-1(qx36).* At least 15 animals (non-transgenic and transgenic) from each independent transgenic line were scored for all lines obtained as indicated in parentheses. Rescue activity was determined by examining the intestine vacuolation phenotype when *chat-1* expression was driven by the *vha-6* promoter (indicated by asterisks); whereas both PS asymmetry and intestine vacuolation phenotypes were scored when *chat-1* expression was controlled by the endogenous promoter. The *chat-1* gene structure is shown with filled boxes representing the exons and thin lines indicating the introns. The arrows pointing away from the 3′ exons delineate the direction of transcription. Three different transcripts of *chat-1* gene are predicted due to alternative splicing. The positions of the intragenic mutation identified in the *qx36* mutant and the genomic deletion in the *chat-1* deletion mutant *ok1681* are also indicated. (B–G) Fluorescent images of *chat-1(qx36)* mutants expressing ssGFP::Lact-C2 driven by heat-shock promoters (P*_hsp_*ssGFP::Lact-C2) and/or P*_chat-1_chat-1a* (B, C) or P*_chat-1_chat-1b* (D, E) or P*_hsp_chat-1c* (F, G). Expression of *chat-1a* but not *chat-1b* or *chat-1c* rescued the PS asymmetry and intestinal vacuolation phenotypes of *chat-1(qx36)* mutants. (H–K) DIC images of the intestine in *tat-1(qx30)* (H) and *chat-1(qx36)* (J) mutants with or without overexpression of *tat-1* (I) or *chat-1* (K) driven by the intestine-specific promoter *vha-6*. The intestinal vacuolation phenotype was rescued by expressing *tat-1* or *chat-1* specifically in the intestine. Scale bars: 5 µm. (L) Phylogenetic tree of yeast, *C. elegans*, and human CDC50 family proteins. Sequences of CDC50 family proteins from yeast, *C. elegans*, and human were compared using the CLUSTALW program. The phylogenetic tree was generated based on the multiple sequence alignment and is shown as a Neighbour-Joining Tree with branch length.
(0.49 MB PDF)

Figure S3 TAT-1 and CHAT-1 are co-dependent for exiting the ER. (A) TAT-1 and CHAT-1 colocalize to plasma membranes and intracellular vesicular and tubular structures. Confocal fluorescent images of wild-type intestine expressing TAT-1::YFP/CHAT-1 and stained with anti-CHAT-1 antibodies. The colocalization of TAT-1 and CHAT-1 was observed on plasma membranes (arrowheads) and intracellular tubular structures (arrows).(B-E)TAT-1 and CHAT-1 are dependent on each other to exit the ER. Fluorescent images of wild-type (B, D), *chat-1(qx36)* (C) and *tat-1(qx30)* (E) intestine carrying both TAT-1::GFP and mCHERRY::TRAM (an ER marker), driven by the *tat-1* and *ges-1* promoters, respectively (B, C), or expressing both CHAT-1::GFP and mCHERRY::TRAM controlled by the *vha-6* and *ges-1* promoters, respectively (D, E). TAT-1::GFP associates with plasma membranes in wild type (B, arrows) but accumulates in the ER in *chat-1(qx36)* mutants (C). Similar accumulation of CHAT-1 in the ER, as indicated by its colocalization with TRAM, was also observed in *tat-1(qx30)* mutants (E). Scale bars: 5 µm.
(0.43 MB PDF)

Figure S4 *tat-1* and *chat-1* mutants accumulate abnormal vacuoles with mixed endolysosomal identities in the intestine. (A–C) DIC images of the intestine in wild type (A), *tat-1(qx30)* (B) and *chat-1(qx36)* (C). Abnormal vacuoles are arrowed. (D-R) Confocal fluorescent images of the intestine in wild type (D, G, J, M, P), *tat-1(qx30)* (E, H, K, N, Q) and *chat-1(qx36)* (F, I, L, O, R) that express GFP::RAB-5 (D–F), GFP::RAB-7 (G-I), GFP::RAB-10 (J–L), GFP::RAB-11 (M–O) or LMP-1::GFP (P–R). The abnormal vacuoles appear to be heterogeneous as they are labeled by markers of different endolysosomal compartments (arrows). Scale bars: 5 µm.
(0.48 MB PDF)

Figure S5 Yolk degradation is affected in *tat-1* and *chat-1* mutants. (A–F) Confocal fluorescent images of intestine in wild type (A, D), *tat-1(qx30)* (B, E) and *chat-1(qx36)* (C, F) stained with anti-RAB-7 (A–C) or anti-RME-1 (D–F) antibodies. In *tat-1* and *chat-1* mutants, aggregation of RAB-7-positive structures was observed (arrows); RME-1-positive vesicles disappear from basolateral membranes (arrowheads), and RME-1 either diffuses or forms aggregated structures (arrows) in the cytoplasm. The non-specific staining of apical membranes by anti-RAB-7 antibodies is marked by asterisks. (G–I) Confocal fluorescent images of 4-fold stage embryos in wild type (G), *tat-1(qx30)* (H) and *chat-1(qx36)* (I) expressing VIT-2::GFP and stained by anti-GFP antibodies. DAPI staining was also included to show nuclei in each embryo. VIT-2::GFP fluorescence and anti-GFP staining were observed in *tat-1(qx30)* and *chat-1(qx36)* but not wild-type embryos. (J–L) Confocal fluorescent images of intestine in wild type (J), *tat-1(qx30)* (K) and *chat-1(qx36)* (L) expressing VIT-2::GFP and stained by anti-GFP antibodies. Animals were aged for 60 h post L4/Adult molt before examination. *tat-1* and *chat-1* intestines accumulate large numbers of yolk granules (arrows). Scale bars: 5 µm.
(0.72 MB PDF)

Figure S6 *tat-1* and *chat-1* mutants contain reduced number of mature lysosomes. (A–F) ER and Golgi markers appear to be normal in *tat-1* and *chat-1* mutants. Confocal fluorescent images of intestine in wild type (A, D), *tat-1(qx30)* (B, E) and *chat-1(qx36)* (C, F) that express MANS::mCHERRY (A–C) or mCHERRY::TRAM (D–F) are shown. (G–I) DIC and confocal fluorescent images of intestine stained by Lyostracker Red in wild type (G), *tat-1(qx30)* (H) and *chat-1(qx36)* (I). The abnormal vacuoles (indicated by asterisks) were not stained by Lysotracker Red. (J) Quantification of Lysotracker Red-positive structures as shown in (G–I). Data are shown as mean numbers of labeled structures ± SEM. *P<1.4×10-12. (K–M) Confocal fluorescent images of hypodermis in wild type (K), *tat-1(qx30)* (L) and *chat-1(qx36)* (M) stained by Lysotracker Red. *tat-1(qx30)* and *chat-1(qx36)* mutants accumulate large acidified compartments positive for Lysotracker Red (arrows) in hypodermal cells. Scale bars: 5 µm.
(0.56 MB PDF)

Figure S7 TAT-1/CHAT-1 associates with tubular membrane structures. (A, B) Confocal fluorescent images of wild-type intestine expressing both TAT-1::GFP and CHAT-1 taken at top (A) and medial (B) focus planes. TAT-1::GFP driven by the *tat-1* promoter (P*_tat-1_*TAT-1::GFP) displayed a vesicular (arrows) and tubular (arrowheads) staining pattern. CHAT-1 controlled by the *vha-6* promoter (P*_vha-6_*CHAT-1) is included to ensure efficient ER exit of TAT-1::GFP.(C–E) Confocal fluorescent images of wild-type (C), *rme-1(b1045)* (D), *rab-10* RNAi (E) intestine expressing CHAT-1::GFP and mCHERRY::RAB-11. Tubular structures labeled by CHAT-1 and RAB-11 (arrows) became further extended in *rme-1(b0145)* mutants (E), but were abolished by *rab-10* RNAi (F). Scale bars: 5 µm.
(0.62 MB PDF)

Figure S8 PS appears on the cytosolic leaflet of plasma membranes and endocytic vesicles. (A–C) DIC and confocal fluorescent images of the intestine in wild-type (A), *tat-1(qx30)* (B) and *chat-1(qx36)* (C) animals expressing GFP::Lact-C2 (P*_ges-1_*GFP::Lact-C2). GFP::Lact-C2 stained both plasma membranes and surfaces of intracellular vesicles in wild-type intestine (A), but labeled abnormal vacuoles in *tat-1 (qx30)* (B) and *chat-1(qx36)* (C) mutants. GFP::Lact-C2-positive plasma membranes and abnormal vacuoles are indicated by arrows and arrowheads respectively. (D–E) PS appears on the cytosolic leaflet of endosome and lysosome membranes in coelomocytes. DIC and fluorescent images of wild-type coelomocytes coexpressing GFP::Lact-C2 with RME-8::mRFP (D) or mCHERRY::CUP-5 (E) are shown. GFP::Lact-C2 expressed specifically in coelomocytes (P*_unc-122_*GFP::Lact-C2) labeled plasma membranes (arrowhead) and the surfaces of both endosomes (stained by RME-8::mRFP; arrows in panel D) and lysosomes (marked by mCHERRY::CUP-5; arrows in panel E). (F–G) ssCHERRY endocytosed by coelomocytes accumulates in lysosomes. DIC and fluorescent images of wild-type coelomocytes carrying both ssCHERRY driven by heat-shock promoters (P*_hsp_*ssCHERRY) and RME-8::GFP (F) or LMP-1::GFP (G) are shown. Secreted CHERRY was endocytosed by coelomocytes from the body cavity and accumulated in lysosomes that are surrounded by LMP-1::GFP (G) (arrows), but not in endosomes that are labeled by RME-8::GFP (F) (arrowheads). (H–J) ssGFP::Lact-C2(AAA) fails to label endomembranes in wild-type, *tat-1* or *chat-1* coelomocytes. DIC and fluorescent images of wild-type (H), *tat-1(qx30)* (I) and *chat-1(qx36)* (J) coelomocytes expressing both ssGFP::Lact-C2(AAA) driven by the *myo-3* promoter (P*_myo3_*ssGFP::Lact-C2(AAA) and ssCHERRY controlled by heat-shock promoters (P*_hsp_*ssCHERRY) are shown. Secreted GFP::Lact-C2(AAA) and CHERRY were endocytosed by coelomocytes and accumulated in lysosomes (arrowheads). ssGFP::Lact-C2(AAA) was either absent or displayed a faint and diffuse pattern in endosomes of wild-type (H), *tat-1(qx30)* (I) and *chat-1(qx36)* (J) coelomocytes (arrows). Scale bars in all panels: 5 µm.
(0.42 MB PDF)

Table S1 Primers used for plasmid construction. Restriction sites are boxed; each site has two protective 5′ nucleotides. The specific reporter names are shown in the “Notes” column for primers that were used for PCR amplification but not individually indicated in the “Plasmid construction” section. Primers for *tat-1* and *chat-1* RNAi are also included. The sequences corresponding to T7 or SP6 are underlined.
(0.08 MB DOC)
