# Supplementary figures and images for: Endocytic Sorting and Recycling Require Membrane Phosphatidylserine Asymmetry Maintained by TAT-1/CHAT-1
Source: PLoS Genet. 2010 Dec 9;6(12):e1001235. doi: 10.1371/journal.pgen.1001235 (PMC3000356; doi:10.1371/journal.pgen.1001235)

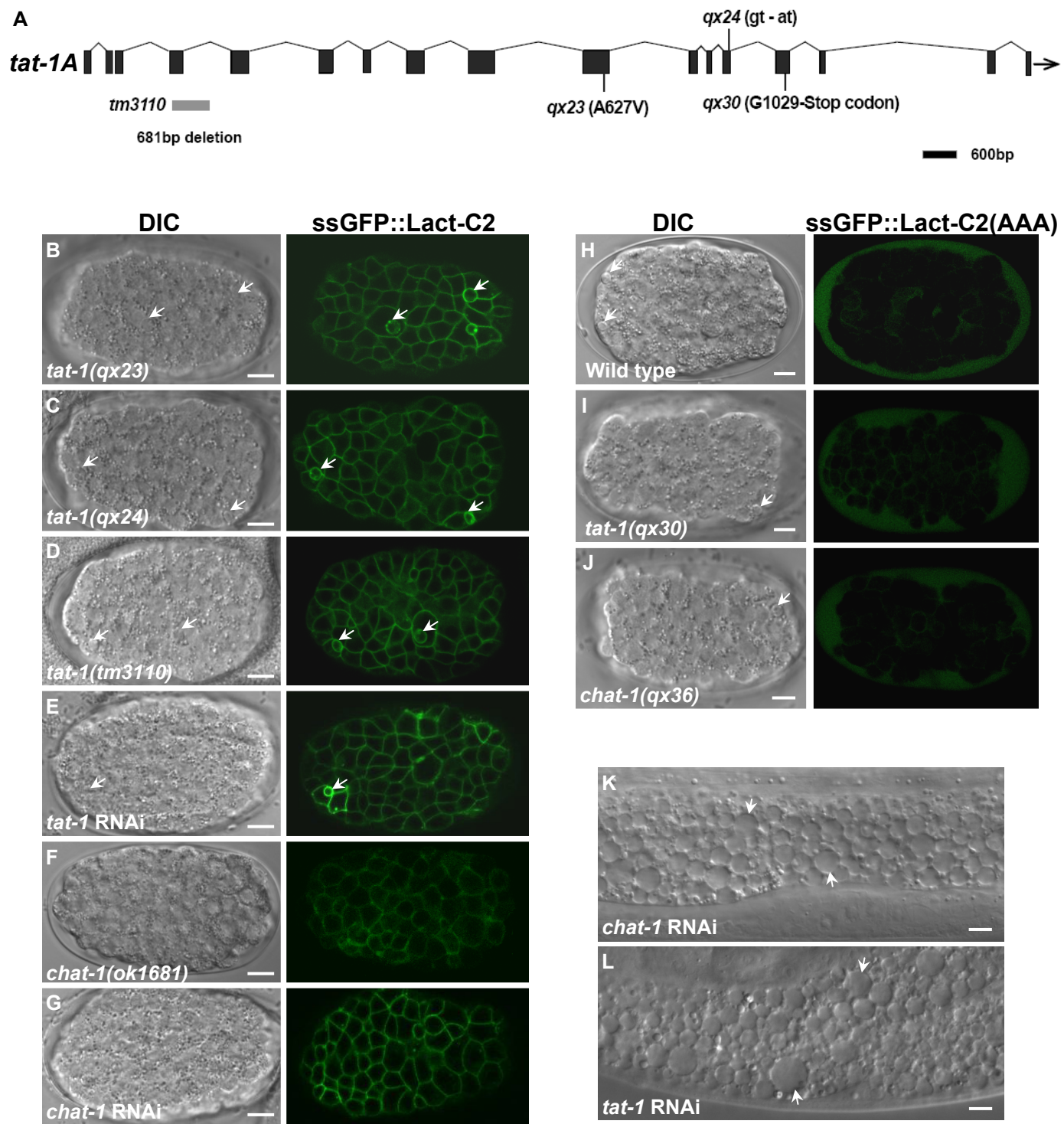

Figure S1

Supplement: Figure S1 — TAT-1 and CHAT-1 are required for maintaining plasma membrane PS asymmetry. (A)Schematic diagram of the C. elegans tat-1 gene. Filled boxes represent exons; thin lines are introns. The arrow shows the direction of transcription. The positions of the four tat-1 mutations (one deletion and three intragenic mutations) are indicated. (B–G) DIC and confocal fluorescent images of various tat-1(lf) and chat-1(lf) embryos expressing the biosensor ssGFP::Lact-C2 driven by heat-shock promoters (PhspssGFP::Lact-C2). Arrows indicate apoptotic cells surrounded by ssGFP::Lact-C2. (H-J) DIC and confocal fluorescent images of wild-type (H), tat-1(qx30) (I) and chat-1(qx36) (J) embryos expressing a secreted biosensor GFP::Lact-C2(AAA) driven by heat-shock promoters. GFP::Lact-C2(AAA) was secreted but failed to label the surfaces of either living or dying cells (indicated by arrows) in wild-type, tat-1(qx30) or chat-1(qx36) embryos. (K–L) Inactivation of tat-1 and chat-1 by RNAi results in abnormal vacuoles in the intestine. DIC images of the intestine of chat-1(RNAi) (K) and tat-1(RNAi) (L) animals are shown. Arrows indicate abnormal vacuoles. Scale bars: 5 µm. (1.29 MB PDF) [file pgen.1001235.s001.pdf]

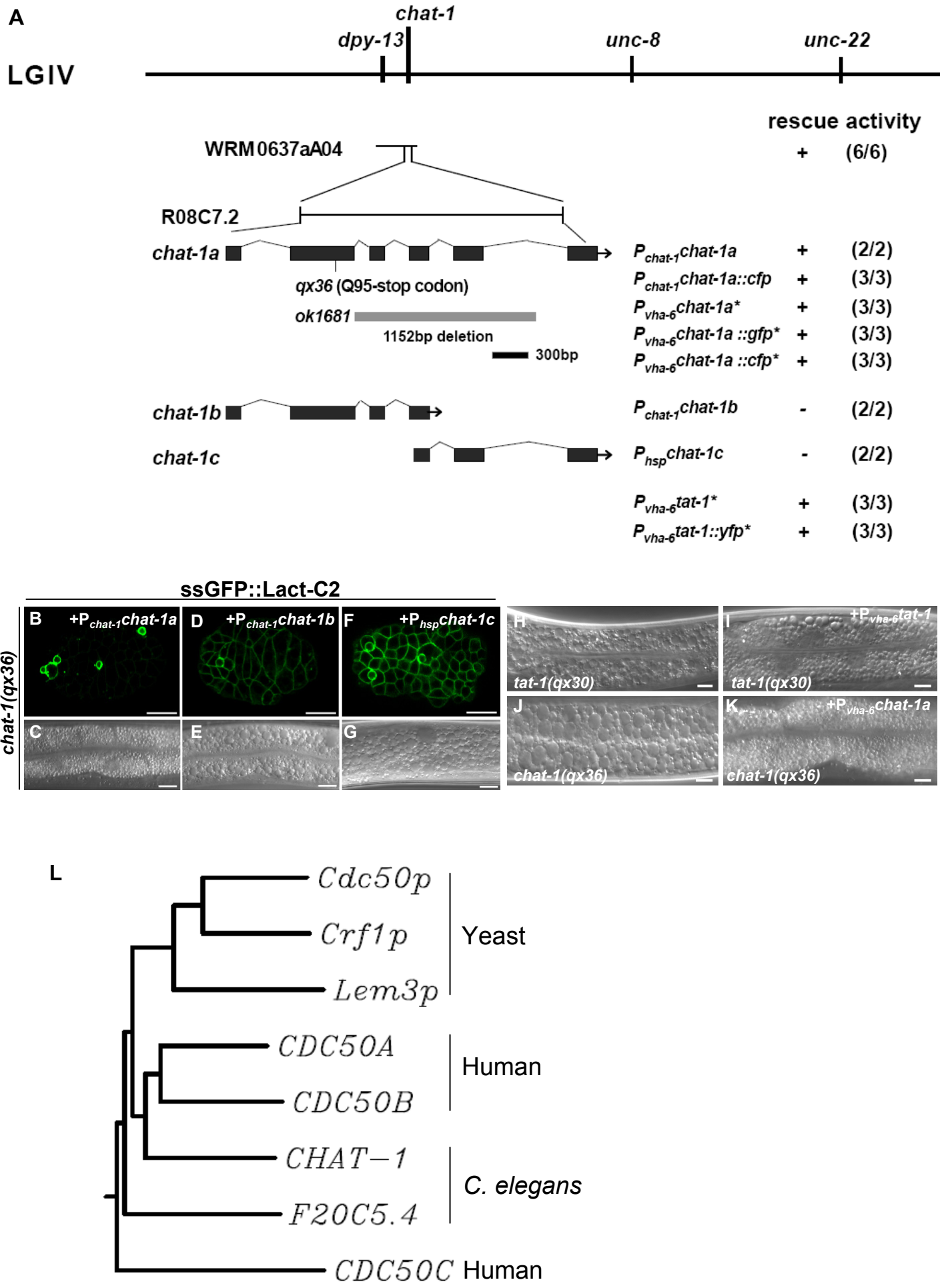

Figure S2

Supplement: Figure S2 — Molecular cloning of chat-1. (A) Cloning of chat-1. The top bar indicates the genetic map of the chat-1 genomic region and the lower panels show the rescue of chat-1(qx36). At least 15 animals (non-transgenic and transgenic) from each independent transgenic line were scored for all lines obtained as indicated in parentheses. Rescue activity was determined by examining the intestine vacuolation phenotype when chat-1 expression was driven by the vha-6 promoter (indicated by asterisks); whereas both PS asymmetry and intestine vacuolation phenotypes were scored when chat-1 expression was controlled by the endogenous promoter. The chat-1 gene structure is shown with filled boxes representing the exons and thin lines indicating the introns. The arrows pointing away from the 3′ exons delineate the direction of transcription. Three different transcripts of chat-1 gene are predicted due to alternative splicing. The positions of the intragenic mutation identified in the qx36 mutant and the genomic deletion in the chat-1 deletion mutant ok1681 are also indicated. (B–G) Fluorescent images of chat-1(qx36) mutants expressing ssGFP::Lact-C2 driven by heat-shock promoters (PhspssGFP::Lact-C2) and/or Pchat-1chat-1a (B, C) or Pchat-1chat-1b (D, E) or Phspchat-1c (F, G). Expression of chat-1a but not chat-1b or chat-1c rescued the PS asymmetry and intestinal vacuolation phenotypes of chat-1(qx36) mutants. (H–K) DIC images of the intestine in tat-1(qx30) (H) and chat-1(qx36) (J) mutants with or without overexpression of tat-1 (I) or chat-1 (K) driven by the intestine-specific promoter vha-6. The intestinal vacuolation phenotype was rescued by expressing tat-1 or chat-1 specifically in the intestine. Scale bars: 5 µm. (L) Phylogenetic tree of yeast, C. elegans, and human CDC50 family proteins. Sequences of CDC50 family proteins from yeast, C. elegans, and human were compared using the CLUSTALW program. The phylogenetic tree was generated based on the multiple sequence alignment and is s [file pgen.1001235.s002.pdf]

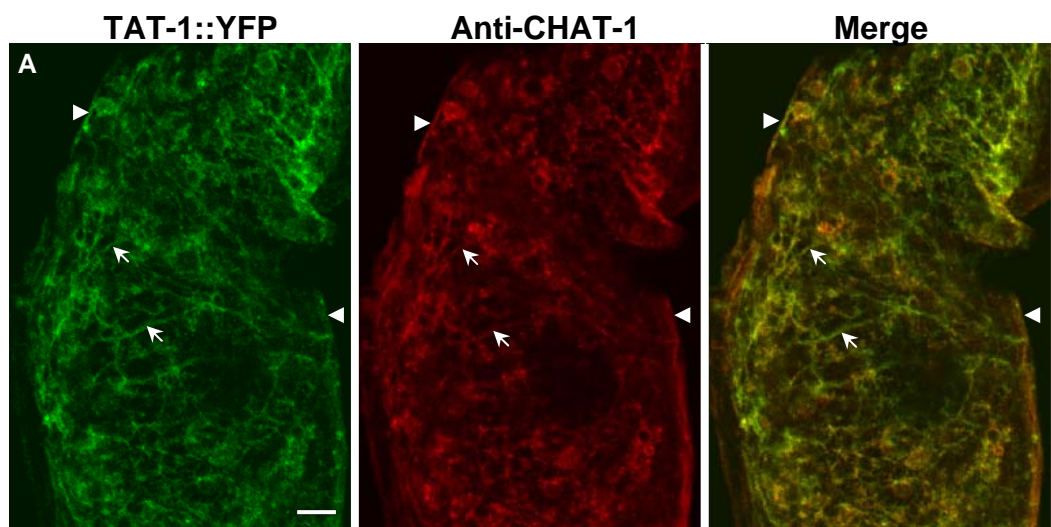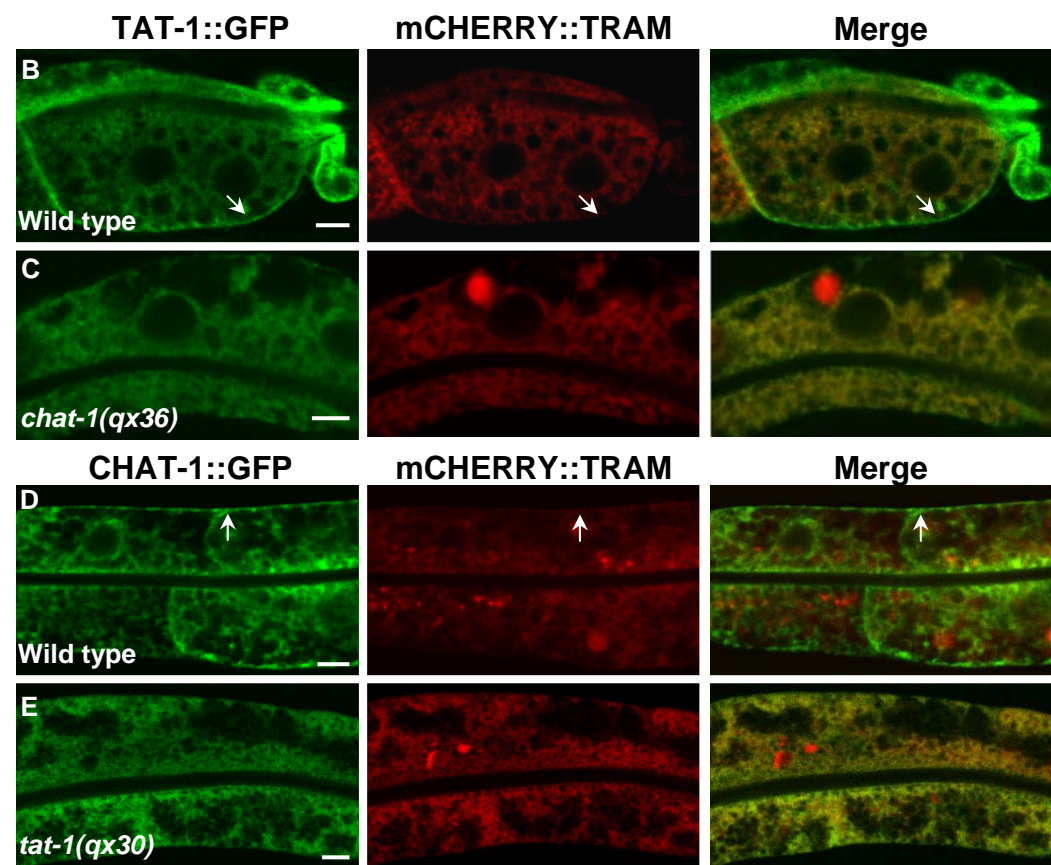

Figure S3

Supplement: Figure S3 — TAT-1 and CHAT-1 are co-dependent for exiting the ER. (A) TAT-1 and CHAT-1 colocalize to plasma membranes and intracellular vesicular and tubular structures. Confocal fluorescent images of wild-type intestine expressing TAT-1::YFP/CHAT-1 and stained with anti-CHAT-1 antibodies. The colocalization of TAT-1 and CHAT-1 was observed on plasma membranes (arrowheads) and intracellular tubular structures (arrows).(B-E)TAT-1 and CHAT-1 are dependent on each other to exit the ER. Fluorescent images of wild-type (B, D), chat-1(qx36) (C) and tat-1(qx30) (E) intestine carrying both TAT-1::GFP and mCHERRY::TRAM (an ER marker), driven by the tat-1 and ges-1 promoters, respectively (B, C), or expressing both CHAT-1::GFP and mCHERRY::TRAM controlled by the vha-6 and ges-1 promoters, respectively (D, E). TAT-1::GFP associates with plasma membranes in wild type (B, arrows) but accumulates in the ER in chat-1(qx36) mutants (C). Similar accumulation of CHAT-1 in the ER, as indicated by its colocalization with TRAM, was also observed in tat-1(qx30) mutants (E). Scale bars: 5 µm. (0.43 MB PDF) [file pgen.1001235.s003.pdf]

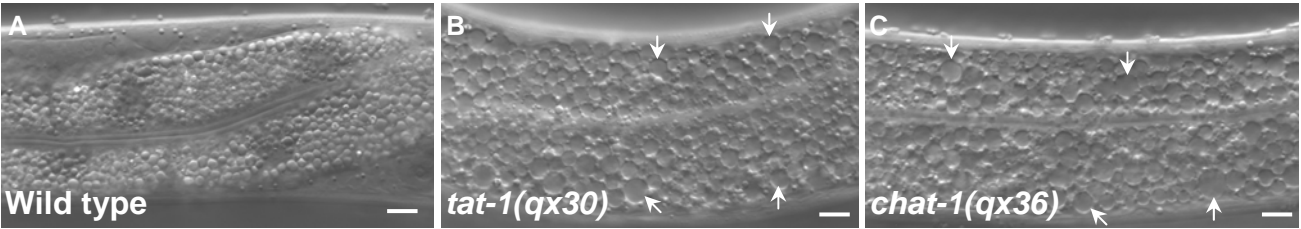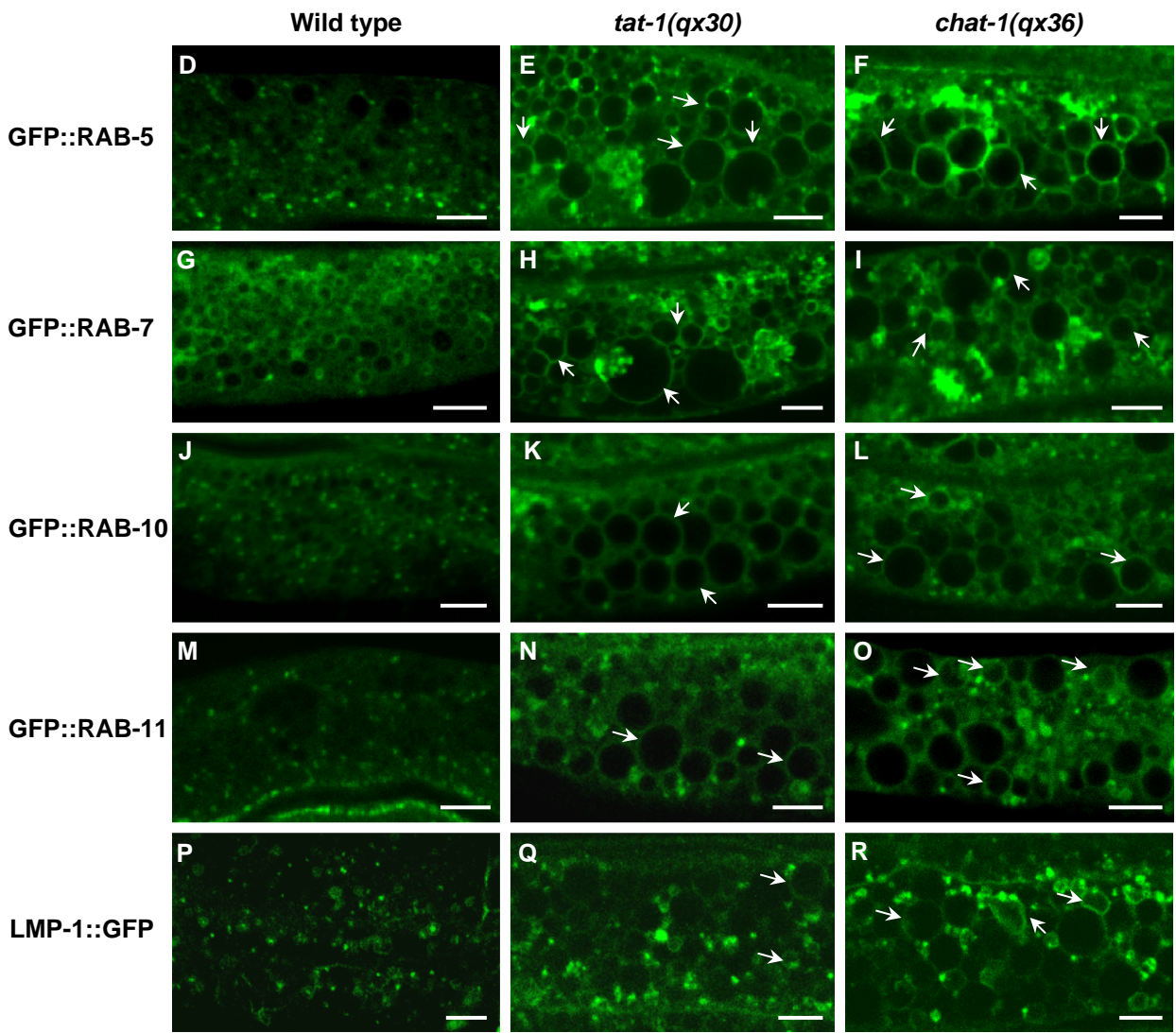

Figure S4

Supplement: Figure S4 — tat-1 and chat-1 mutants accumulate abnormal vacuoles with mixed endolysosomal identities in the intestine. (A–C) DIC images of the intestine in wild type (A), tat-1(qx30) (B) and chat-1(qx36) (C). Abnormal vacuoles are arrowed. (D-R) Confocal fluorescent images of the intestine in wild type (D, G, J, M, P), tat-1(qx30) (E, H, K, N, Q) and chat-1(qx36) (F, I, L, O, R) that express GFP::RAB-5 (D–F), GFP::RAB-7 (G-I), GFP::RAB-10 (J–L), GFP::RAB-11 (M–O) or LMP-1::GFP (P–R). The abnormal vacuoles appear to be heterogeneous as they are labeled by markers of different endolysosomal compartments (arrows). Scale bars: 5 µm. (0.48 MB PDF) [file pgen.1001235.s004.pdf]

Wild type

*tat-1(qx30)**chat-1(qx36)*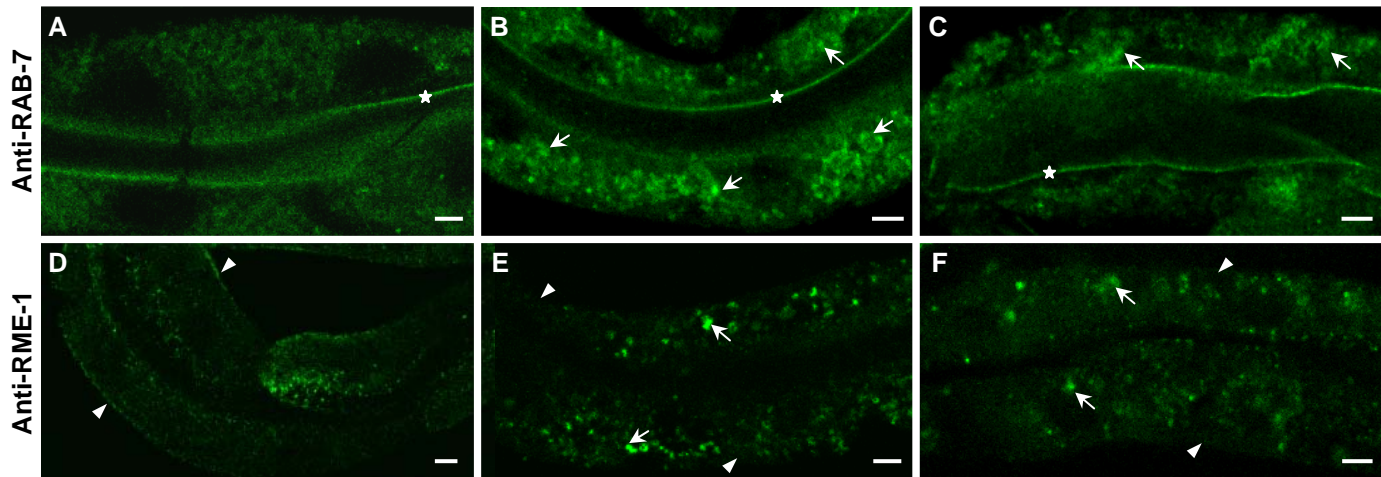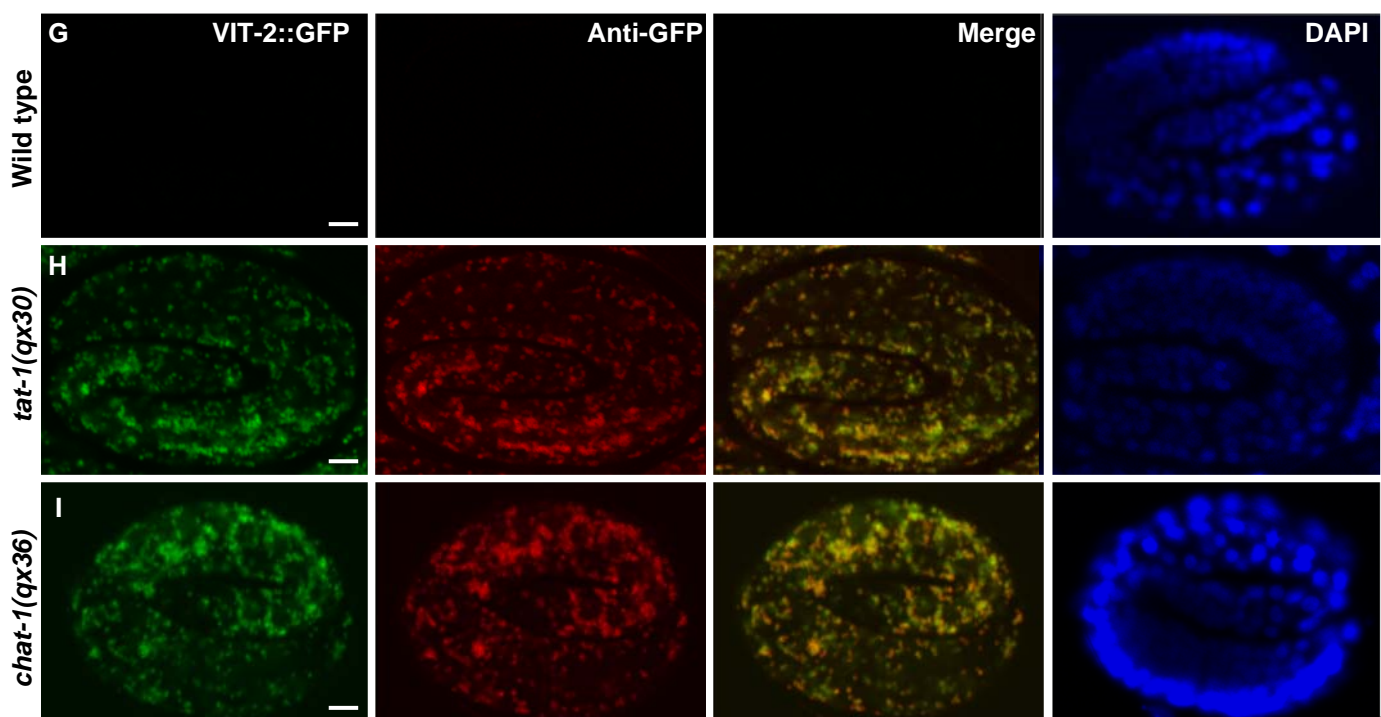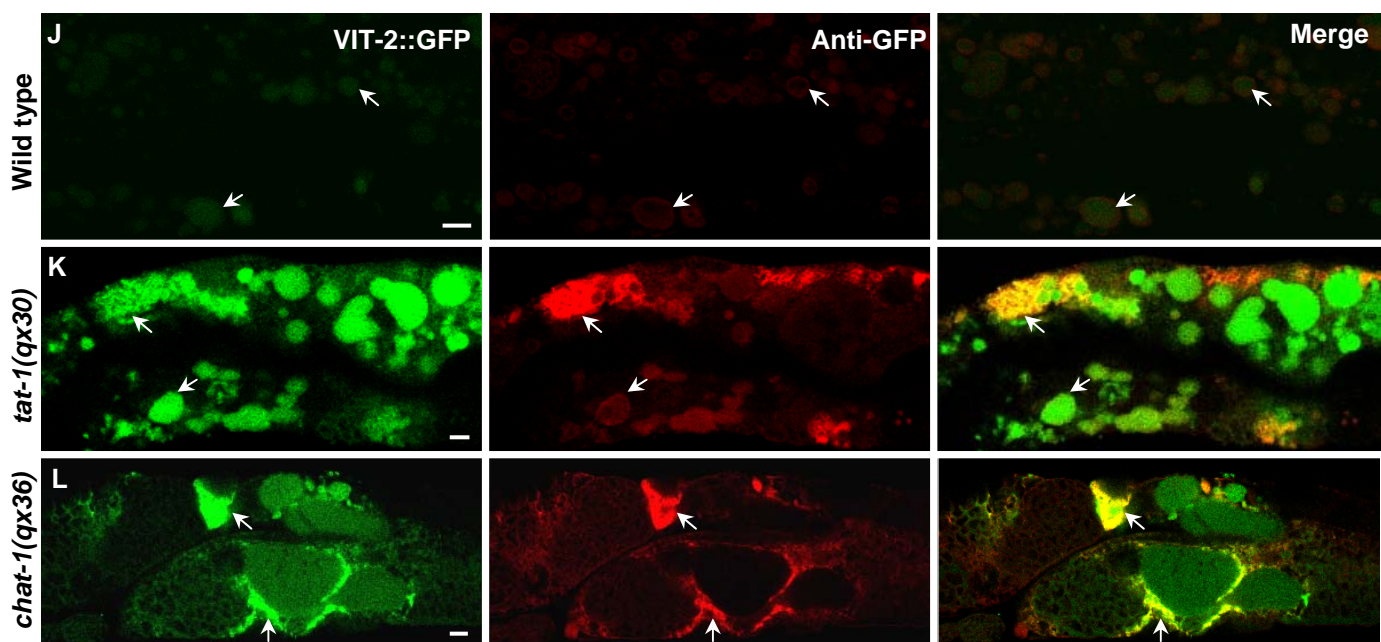

Figure S5

Supplement: Figure S5 — Yolk degradation is affected in tat-1 and chat-1 mutants. (A–F) Confocal fluorescent images of intestine in wild type (A, D), tat-1(qx30) (B, E) and chat-1(qx36) (C, F) stained with anti-RAB-7 (A–C) or anti-RME-1 (D–F) antibodies. In tat-1 and chat-1 mutants, aggregation of RAB-7-positive structures was observed (arrows); RME-1-positive vesicles disappear from basolateral membranes (arrowheads), and RME-1 either diffuses or forms aggregated structures (arrows) in the cytoplasm. The non-specific staining of apical membranes by anti-RAB-7 antibodies is marked by asterisks. (G–I) Confocal fluorescent images of 4-fold stage embryos in wild type (G), tat-1(qx30) (H) and chat-1(qx36) (I) expressing VIT-2::GFP and stained by anti-GFP antibodies. DAPI staining was also included to show nuclei in each embryo. VIT-2::GFP fluorescence and anti-GFP staining were observed in tat-1(qx30) and chat-1(qx36) but not wild-type embryos. (J–L) Confocal fluorescent images of intestine in wild type (J), tat-1(qx30) (K) and chat-1(qx36) (L) expressing VIT-2::GFP and stained by anti-GFP antibodies. Animals were aged for 60 h post L4/Adult molt before examination. tat-1 and chat-1 intestines accumulate large numbers of yolk granules (arrows). Scale bars: 5 µm. (0.72 MB PDF) [file pgen.1001235.s005.pdf]

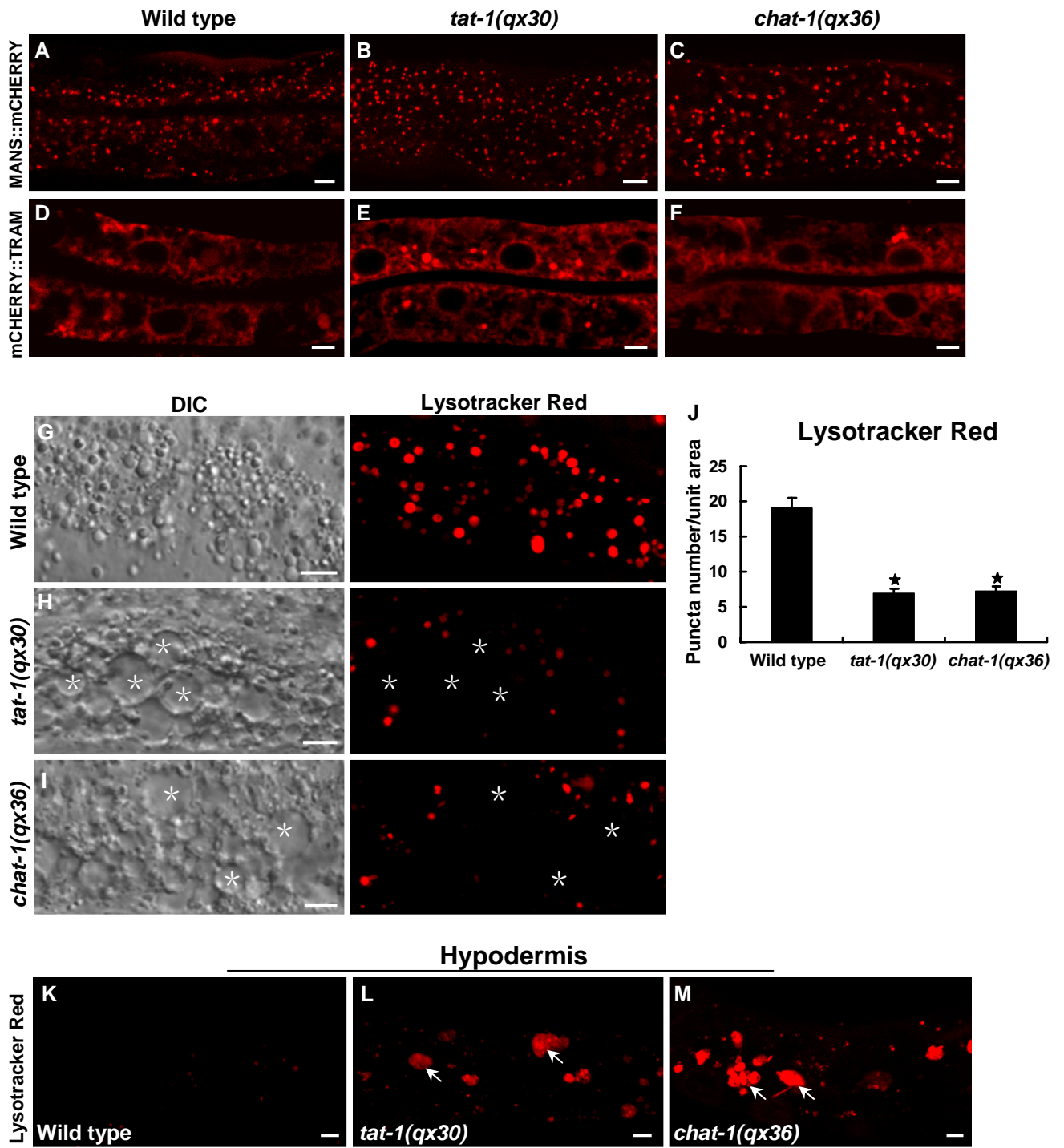

Figure S6

Supplement: Figure S6 — tat-1 and chat-1 mutants contain reduced number of mature lysosomes. (A–F) ER and Golgi markers appear to be normal in tat-1 and chat-1 mutants. Confocal fluorescent images of intestine in wild type (A, D), tat-1(qx30) (B, E) and chat-1(qx36) (C, F) that express MANS::mCHERRY (A–C) or mCHERRY::TRAM (D–F) are shown. (G–I) DIC and confocal fluorescent images of intestine stained by Lyostracker Red in wild type (G), tat-1(qx30) (H) and chat-1(qx36) (I). The abnormal vacuoles (indicated by asterisks) were not stained by Lysotracker Red. (J) Quantification of Lysotracker Red-positive structures as shown in (G–I). Data are shown as mean numbers of labeled structures ± SEM. *P<1.4×10^-12^. (K–M) Confocal fluorescent images of hypodermis in wild type (K), tat-1(qx30) (L) and chat-1(qx36) (M) stained by Lysotracker Red. tat-1(qx30) and chat-1(qx36) mutants accumulate large acidified compartments positive for Lysotracker Red (arrows) in hypodermal cells. Scale bars: 5 µm. (0.56 MB PDF) [file pgen.1001235.s006.pdf]

# TAT-1::GFP

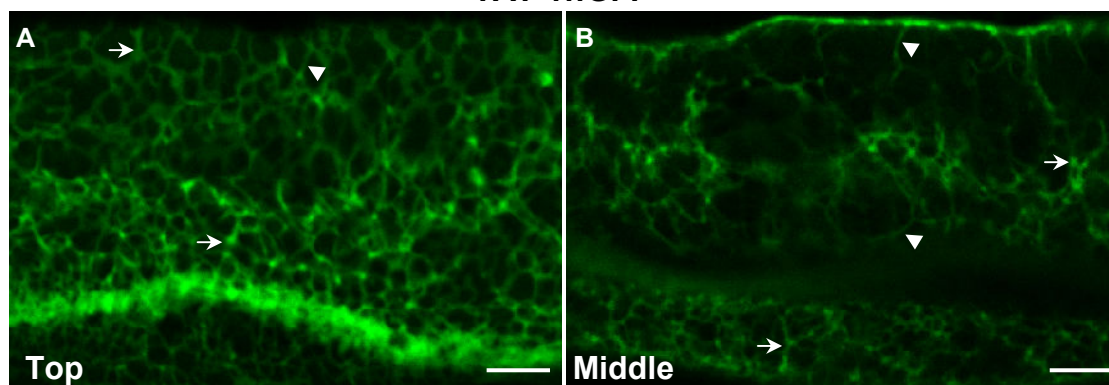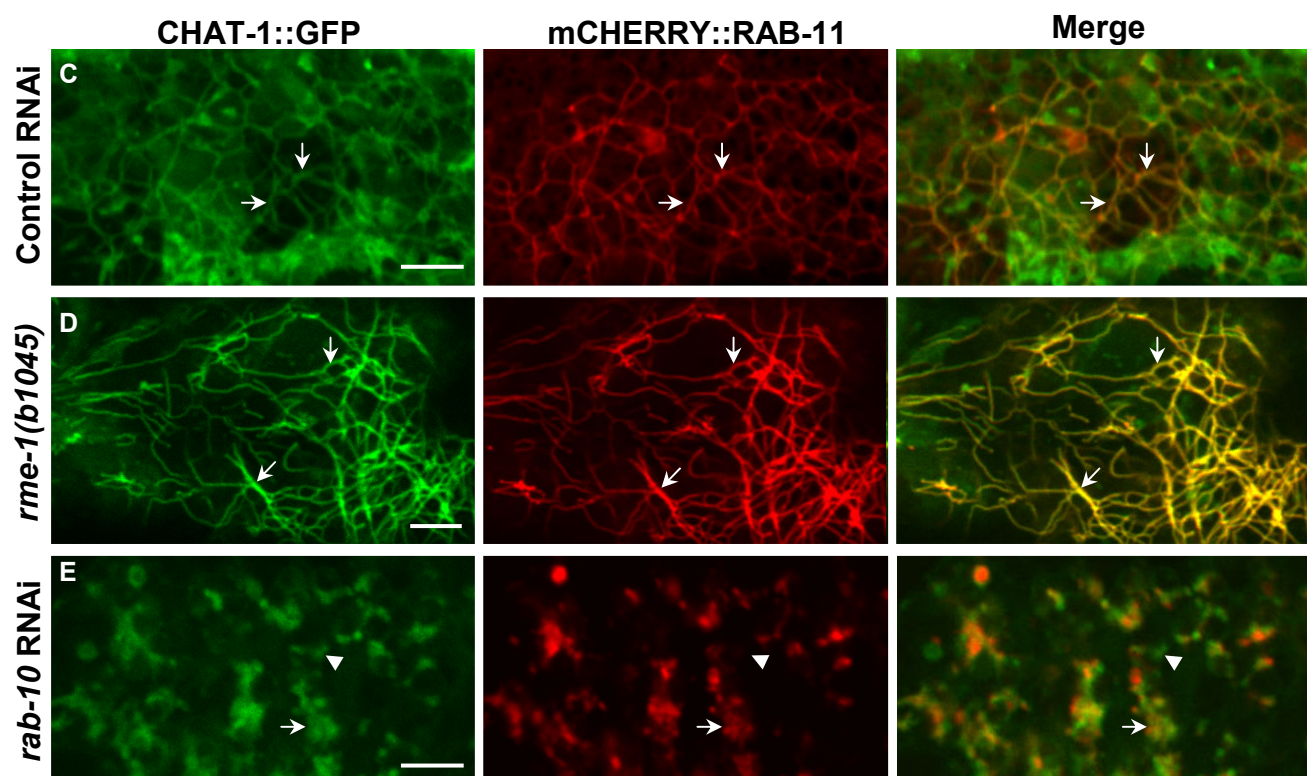

Figure S7

Supplement: Figure S7 — TAT-1/CHAT-1 associates with tubular membrane structures. (A, B) Confocal fluorescent images of wild-type intestine expressing both TAT-1::GFP and CHAT-1 taken at top (A) and medial (B) focus planes. TAT-1::GFP driven by the tat-1 promoter (Ptat-1TAT-1::GFP) displayed a vesicular (arrows) and tubular (arrowheads) staining pattern. CHAT-1 controlled by the vha-6 promoter (Pvha-6CHAT-1) is included to ensure efficient ER exit of TAT-1::GFP.(C–E) Confocal fluorescent images of wild-type (C), rme-1(b1045) (D), rab-10 RNAi (E) intestine expressing CHAT-1::GFP and mCHERRY::RAB-11. Tubular structures labeled by CHAT-1 and RAB-11 (arrows) became further extended in rme-1(b0145) mutants (E), but were abolished by rab-10 RNAi (F). Scale bars: 5 µm. (0.62 MB PDF) [file pgen.1001235.s007.pdf]

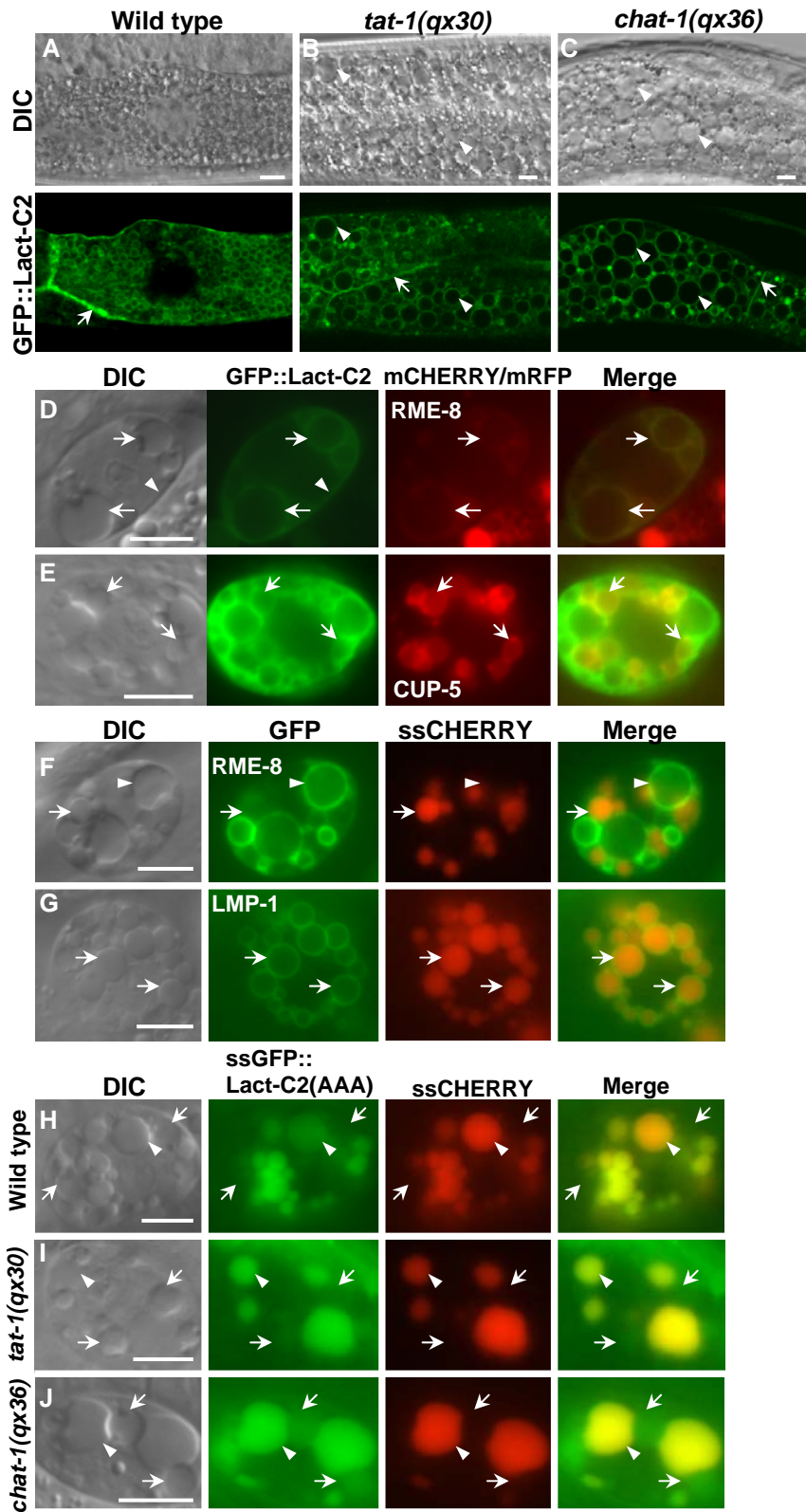

Figure S8

Supplement: Figure S8 — PS appears on the cytosolic leaflet of plasma membranes and endocytic vesicles. (A–C) DIC and confocal fluorescent images of the intestine in wild-type (A), tat-1(qx30) (B) and chat-1(qx36) (C) animals expressing GFP::Lact-C2 (Pges-1GFP::Lact-C2). GFP::Lact-C2 stained both plasma membranes and surfaces of intracellular vesicles in wild-type intestine (A), but labeled abnormal vacuoles in tat-1 (qx30) (B) and chat-1(qx36) (C) mutants. GFP::Lact-C2-positive plasma membranes and abnormal vacuoles are indicated by arrows and arrowheads respectively. (D–E) PS appears on the cytosolic leaflet of endosome and lysosome membranes in coelomocytes. DIC and fluorescent images of wild-type coelomocytes coexpressing GFP::Lact-C2 with RME-8::mRFP (D) or mCHERRY::CUP-5 (E) are shown. GFP::Lact-C2 expressed specifically in coelomocytes (Punc-122GFP::Lact-C2) labeled plasma membranes (arrowhead) and the surfaces of both endosomes (stained by RME-8::mRFP; arrows in panel D) and lysosomes (marked by mCHERRY::CUP-5; arrows in panel E). (F–G) ssCHERRY endocytosed by coelomocytes accumulates in lysosomes. DIC and fluorescent images of wild-type coelomocytes carrying both ssCHERRY driven by heat-shock promoters (PhspssCHERRY) and RME-8::GFP (F) or LMP-1::GFP (G) are shown. Secreted CHERRY was endocytosed by coelomocytes from the body cavity and accumulated in lysosomes that are surrounded by LMP-1::GFP (G) (arrows), but not in endosomes that are labeled by RME-8::GFP (F) (arrowheads). (H–J) ssGFP::Lact-C2(AAA) fails to label endomembranes in wild-type, tat-1 or chat-1 coelomocytes. DIC and fluorescent images of wild-type (H), tat-1(qx30) (I) and chat-1(qx36) (J) coelomocytes expressing both ssGFP::Lact-C2(AAA) driven by the myo-3 promoter (Pmyo3ssGFP::Lact-C2(AAA) and ssCHERRY controlled by heat-shock promoters (PhspssCHERRY) are shown. Secreted GFP::Lact-C2(AAA) and CHERRY were endocytosed by coelomocytes and accumulated in lysosomes (arrowheads). ssGFP::Lact-C2(AAA) was either absent or [file pgen.1001235.s008.pdf]
