# Supplementary material for: Endocytic Sorting and Recycling Require Membrane Phosphatidylserine Asymmetry Maintained by TAT-1/CHAT-1
Source: PLoS Genet. 2010 Dec 9;6(12):e1001235. doi: 10.1371/journal.pgen.1001235 (PMC3000356; doi:10.1371/journal.pgen.1001235)
Supplement: Table S1 — Primers used for plasmid construction. Restriction sites are boxed; each site has two protective 5′ nucleotides. The specific reporter names are shown in the “Notes” column for primers that were used for PCR amplification but not individually indicated in the “Plasmid construction” section. Primers for tat-1 and chat-1 RNAi are also included. The sequences corresponding to T7 or SP6 are underlined. (0.08 MB DOC) [file pgen.1001235.s009.doc]

**Table S1.** Primers used for plasmid construction

| **Primer** | **Sequence** | **Notes** |
| --- | --- | --- |
| PXCW241 | gcggtaccatggccgcccgaaacgcaggaacc | GFP::RAB-5  mCHERRY::RAB-5 |
| PXCW242 | gccccgggttatttacagcatgaacccttttg | mCHERRY::RAB-5 |
| PXCW243 | gcggtaccttatttacagcatgaacccttttg | GFP::RAB-5 |
| PXCW244 | gcggtacc atgtcgggaaccagaaagaaggcg | GFP::RAB-7  mCHERRY::RAB-7 |
| PXCW245 | gcgatatcttaacaattgcatcccgaattctgc | mCHERRY::RAB-7 |
| PXCW246 | gcggtacctaacaattgcatcccgaattctgc | GFP::RAB-7 |
| PBHC123 | ccggtaccatgcccacagaggcaagagataataatc |  |
| PBHC124 | ccggtaccttatcgtccagtcggtttttctctagtag |  |
| PBHC144 | ccgatatctgcactgaacccctaggcctgaag |  |
| PBHC145 | ccgagctcctaacagcccagcagctccactc |  |
| PBHC146 | ccggtacctgcactgaacccctaggcctgaag | GFP::Lact-C2  mCHERRY::Lact-C2 |
| PBHC147 | ccggtaccctaacagcccagcagctccactc |
| PBHC154 | ccgctagcatgcccacagaggcaagagataataatc |  |
| PBHC156 | cctccagctactacaaaaccgcgggcctgagtgcctttagcgcggctccctactacgcacgactgga | GFP::Lact-C2(AAA) |
| PBHC157 | tccagtcgtgcgtagtagggagccgcgctaaaggcactcaggcccgcggttttgtagtagctggagg |
| PBHC171 | gcggtacc atgggctctcgtgacgatgaatac | mCHERRY::RAB-11  GFP::RAB-11 |
| PBHC172 | gcggtacc ttatgggatgcaacactgcttc |
| PBHC204 | cggctagcatgaacgccgttgttgccagtc | GLUT1::GFP  GLUT1::CFP |
| PBHC205 | cggctagctgaacatatccgttttctcatg |
| PWZ263 | tttttatgggttttggtaggtttt |  |
| PWZ512 | gaaacgcgcgagacgaaagggcccgt |  |
| PWZ624 | gacattaggtgtcacacaaaag |  |
| PWZ625 | taaaacctaccaaaacccataaaaa atgcccacagaggcaagagata |  |
| PJCY4 | ccgctagctcgtccagtcggtttttctctagtag |  |
| PJCY6 | gggctagcgtgatgaatttccgagacgttgctc |  |
| PJCY9 | tccccccgggtctaaaccaacttgatatttttgtg |  |
| PJCY16 | ggaagcttgctattgcctagagcttgatttg |  |
| PJCY17 | gggctagctatgctccaaaaagtgtttgac |  |
| PJCY18 | gggctagcatgccgccgagagacgccgttccg |  |
| PJCY20 | gggctagcatggtacccgtgacgactcag |  |
| PJCY21 | gggcatgcccagccggatcgtaatcgaatcctcg |  |
| PJCY34 | gggctagcttagtgatgaatttccgagacgttgctc |  |
| PJCY35 | gggctagcttatgacgtcagtacgttgaatgcgtc |  |
| PYJ7 | catgccatggtcagtgatgaatttccgagacg |  |
| PYJ40 | cggctagcatgggaaaacgcaatttc |  |
| PYJ44 | ggggtaccggcagcggcggcggcggcagcggcggcggcggcagcggcatggttaagccgcaaggaggg |  |
| PYJ45 | ggggtaccttaatttttcttcttcgaatcg |  |
| PYJ53 | cggctagcgccgctgccgccgccgccgctgccgccgccgccgctgccttctttttcttcatcaaaatc |  |
| PYJ55 | cgggtaccatggctcgccgaccgtatgac | mCHERRY::RAB-10  GFP::RAB-10 |
| PYJ56 | cgggtaccctagcagcatcctccactgctac |
| PYZ203 | gcgctagcatgcataaggttttgctggcac |  |
| PYZ206 | gcactagttttttctaccggtaccgtcgacgc |
| PPFG199 | aagggcccgtacggccgactagtagg |  |
| PPFG211 | cgggatccccatcaacttcttcactggt |  |
| PPFG343 | ctagctagcatggactcgggccgggacttcc |  |
| PPFG344 | gggatatcttagatgttgagctccttcaggaag |  |
| PLNY9 | cactagtaatacgactcactatagggttgttcccgagaacaaggatggtc | *tat-1* RNAi (T7) |
| PLNY10 | cactagatttaggtgacactatagaactcctgaagcttatcctcaatcgc | *tat-1* RNAi (SP6) |
| PLNY61 | cactagtaatacgactcactataggggagagacgccgttccgacgtcgacgc | *chat-1* RNAi (T7) |
| PLNY62 | cactagatttaggtgacactatagaacgagcacaatggccagggatccgag | *chat-1* RNAi (SP6) |

Restriction sites are boxed; each site has two protective 5’ nucleotides. The specific reporter names are shown in the ‘Notes’ column for primers that were used for PCR amplification but not individually indicated in the “Plasmid Construction” section. Primers for *tat-1* and *chat-1* RNAi are also included. The sequences corresponding to T7 or SP6 are underlined.
